# Irradiation-induced grain boundary facet motion: In situ observations and atomic-scale mechanisms

**DOI:** 10.1126/sciadv.abn0900

**Published:** 2022-06-10

**Authors:** Christopher M. Barr, Elton Y. Chen, James E. Nathaniel, Ping Lu, David P. Adams, Rémi Dingreville, Brad L. Boyce, Khalid Hattar, Douglas L. Medlin

**Affiliations:** 1 Sandia National Laboratories, Albuquerque, NM 87185, USA.; 2 Sandia National Laboratories, Livermore, CA 94550, USA.

## Abstract

Metals subjected to irradiation environments undergo microstructural evolution and concomitant degradation, yet the nanoscale mechanisms for such evolution remain elusive. Here, we combine in situ heavy ion irradiation, atomic resolution microscopy, and atomistic simulation to elucidate how radiation damage and interfacial defects interplay to control grain boundary (GB) motion. While classical notions of boundary evolution under irradiation rest on simple ideas of curvature-driven motion, the reality is far more complex. Focusing on an ion-irradiated Pt Σ3 GB, we show how this boundary evolves by the motion of 120° facet junctions separating nanoscale {112} facets. Our analysis considers the short- and mid-range ion interactions, which roughen the facets and induce local motion, and longer-range interactions associated with interfacial disconnections, which accommodate the intergranular misorientation. We suggest how climb of these disconnections could drive coordinated facet junction motion. These findings emphasize that both local and longer-range, collective interactions are important to understanding irradiation-induced interfacial evolution.

## Teaser

Microscopy and modeling elucidate short- and long-range mechanisms controlling irradiation-induced motion of grain boundary facets.

## INTRODUCTION

Establishing the mechanisms that underpin microstructural aging in radiation environments poses a fundamental materials research challenge. Understanding these mechanisms, particularly the interplay between irradiation-induced displacement damage and interfacial dynamics, is important because it may suggest strategies for predicting or improving radiation tolerance of materials. As was recently reviewed (*1*), many studies have shown the ability of interfaces to act as defect sinks and mitigate radiation damage when spaced within a few nanometers of each other (*2*, *3*). A range of nanocrystalline metals have shown improvement in radiation tolerance compared with coarse-grained materials, as measured by defect density and defect size. Where this improvement is observed, it is generally associated with the increase in interface density and the high point defect cluster sink efficiency of these interfaces (*4*, *5*). The effectiveness of such interfacially mediated mechanisms depends sensitively on several issues including grain boundary (GB) character (*6*), the specific type of defect cluster (*7*), and other factors including irradiation species, radiation flux, and contamination level (*8*). Temperature can also play an important role. For instance, in comparison to larger-grained material, nanocrystalline Au has shown an increase in defect accumulation rate when subjected to swift-ion irradiation at very low temperatures (15 K), but a decrease in defect accumulation rate when irradiated at room temperature (*9*).

Irradiation is also well known to accelerate the rate of grain coarsening (*10*–*13*). As with thermal coarsening, irradiation-enhanced coarsening is typically considered as a curvature-driven process, with grains evolving under capillary forces to reduce their total interfacial free energy. Irradiation is thought to increase the mobility of the interfaces allowing GB motion to occur at global temperatures well below the thermal onset of coarsening. Such enhancements in mobility have been modeled by accounting for how collisional cascades (*10*) or associated ion-induced thermal events (*11*–*13*) increase atomic jump rates across the interfaces. For instance, Kaoumi *et al*. (*12*) have shown how considerations of the probability of a thermal event intersecting a GB can explain the t^1/3^ kinetics experimentally observed in ion-induced grain coarsening of polycrystalline thin films. More recently, Bufford *et al*. (*13*) have shown that the level of ion irradiation–induced grain mobility can vary for different types of boundaries.

Although grain coarsening and its relation to irradiation-enhanced mobility are well known, what is less clear is how these processes occur at the atomic scale in concert with the local, atomic structure of the boundary. A key question is how irradiation couples with extended interfacial defects, such as disconnections and facet junctions, that can arise at singular interfaces at cusps in the misorientation-inclination energy landscape. For boundary motion under thermal activation, there is growing recognition of the controlling nature of such GB line defects (*14*–*16*), leading one group to suggest that “conventional grain growth theory must be viewed as a simplistic model that does not account for the fact that real materials are crystalline and crystallinity imposes constraints on how GBs move” (*17*). Recent experiments on the thermal evolution of polycrystalline Ni have demonstrated the inadequacy of simple continuum, curvature-based ideas in predicting GB velocity (*18*). These observations further underscore the importance of understanding anisotropy with respect to interfacial misorientation and inclination space as well as the role of line defects, such as disconnections and facet junctions, which are associated with such orientational energy anisotropy. One would expect such considerations also to be critical to understanding GB evolution in an irradiation environment.

In response, we report here on in situ observations and analysis of irradiation-induced GB facet migration in nanocrystalline platinum. We focus specifically on a boundary composed of Σ3{112} facets, an interface that is commonly termed the “incoherent” or “lateral” twin to distinguish it from the “coherent” {111} twin. Σ3{112} boundaries present a useful system for investigating the effects of interfacial anisotropy and line defects on irradiation-induced boundary motion because their baseline structure is well understood for many different face-centered cubic (FCC) metals (*19*–*25*) including Pt (*26*). Moreover, this type of interface is directly relevant to nanotwinned materials because the motion of {112} facets, which occurs more readily than that of {111} facets, is critical to the stability of these nanostructured materials (*27*, *28*).

Prior electron microscopy experiments have already shown that ion irradiation can induce the motion of Σ3{112} facets in nanotwinned FCC metals (*29*–*34*). In contrast to these prior studies, which have concentrated on {112} boundaries forming 90° junctions with {111} coherent twin facets, here we focus on boundaries consisting solely of nanoscale 120°-related {112} facets. Because the adjacent interfaces are all crystallographically equivalent, such a geometry is convenient for a targeted analysis of GB junction effects, as has been done previously to elucidate junction response to thermal excitation (*35*–*37*).

With the aid of time-resolved, in situ observations under conditions of nonimplanting heavy ion irradiation (Au^4+^) combined with ex situ atomic resolution imaging before and after the irradiation, we begin to unravel the process by which such nanofaceted boundaries migrate under an irradiation flux. Atomistic modeling examines the critical role that facet junctions play in the migration process and helps to parse the relative contributions of the local atomic rearrangements due to ion strikes from the defect (interstitial vacancy) generation, damage cascade, and concomitant thermal event, often referred to as a thermal spike. Analysis of the electron microscopic observations provides insight into the defect content of the GB and its relationship to the intergranular misorientation and facet morphology. We discuss these results both in terms of the shorter-range processes leading to GB facet roughening and the longer-range mechanisms governing the coarser scale boundary evolution.

## RESULTS

We begin by discussing our experimental observations of the structure of the faceted Σ3 boundary. After establishing the initial baseline structure of the interface, we then present in situ observations of the boundary evolution under heavy ion irradiation. Last, we compare our observations of the pre- and post-irradiated boundary to quantify the evolution of the facet structure and its connection to interfacial line defects present to accommodate coherency strains at the GB resulting because of its misorientation.

**Fig. 1.**
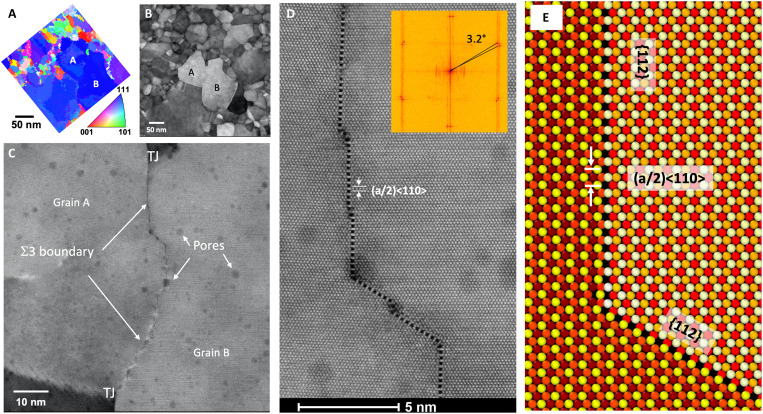
The analyzed GB and its surrounding environment. (**A**) Automated crystal orientation mapping showing the grain orientations in the vicinity of the interface of interest. The boundary of interest separates the two indicated grains, labeled as A and B, at the center of image (**B**) and terminates at triple junctions [labeled TJ in (**C**)]. The boundary is faceted on Σ3 {112} interfaces that intersect at 120°. (**D**) High-angle annular dark field scanning transmission electron microscopy image showing structure at atomic resolution. (**E**) Atomistic model [embedded atom method (EAM)] for the ideal facet and junction structure. Fast Fourier transform analysis of the atomic resolution images [inset in (D)] shows that the grains are rotated by 3.2° from the exact Σ3 orientation.

Our observations were conducted on a nanocrystalline Pt thin film. The thickness of this film was limited to 18 nm to enable suitable electron transmission for high-quality atomic resolution imaging. Figure 1 shows the faceted Σ3 GB investigated in this study and its surrounding environment of nanoscale grains. The imaging here was conducted using automated crystal orientation mapping (ACOM) and high-angle annular dark-field scanning transmission electron microscopy (HAADF-STEM). The as-deposited and thermally annealed film contains some scattered pore-like features (typically about 1 nm in diameter), which are visible as slightly darker regions in the HAADF-STEM images. The boundary of interest separates the two grains indicated at the center of Fig. 1 (A to C). Both these grains are oriented along <111> out of the plane, as confirmed with electron diffraction as part of the ACOM analysis (Fig. 1A) and high-resolution STEM (HRSTEM) imaging (Fig. 1, C and D). The boundary terminates at triple junctions at the top and bottom of the grain. Close inspection of the boundary (Fig. 1C) reveals a nonplanar, faceted morphology that is convex into the right grain (“grain B”). Higher magnification imaging (Fig. 1D) reveals that the facets are aligned along {112}-type planes that intersect at 120° angles. Figure 1E shows an atomistic model of one such {112} facet junction. Along the {112} facets, the periodic array of dark spots that repeat with a period of (a/2)<110> correspond to channels along the <111> direction of the boundary, as indicated in the model. This structural interpretation is consistent with previous high-resolution TEM observations and simulations of {112} facets in <111>-oriented Au Σ3 thin film bicrystals (*38*).

The two grains deviate slightly from the exact 60° Σ3 orientation relationship. Measurements from fast Fourier transforms (FFTs) of the atomically resolved HRSTEM images show an intergranular misorientation about the shared <111> axis of 3.2° from Σ3 (Fig. 1D). As we analyze in greater detail below, this misorientation is accommodated by an array of GB defects that are localized to steps and facet junctions on the boundary.

**Fig. 2.**
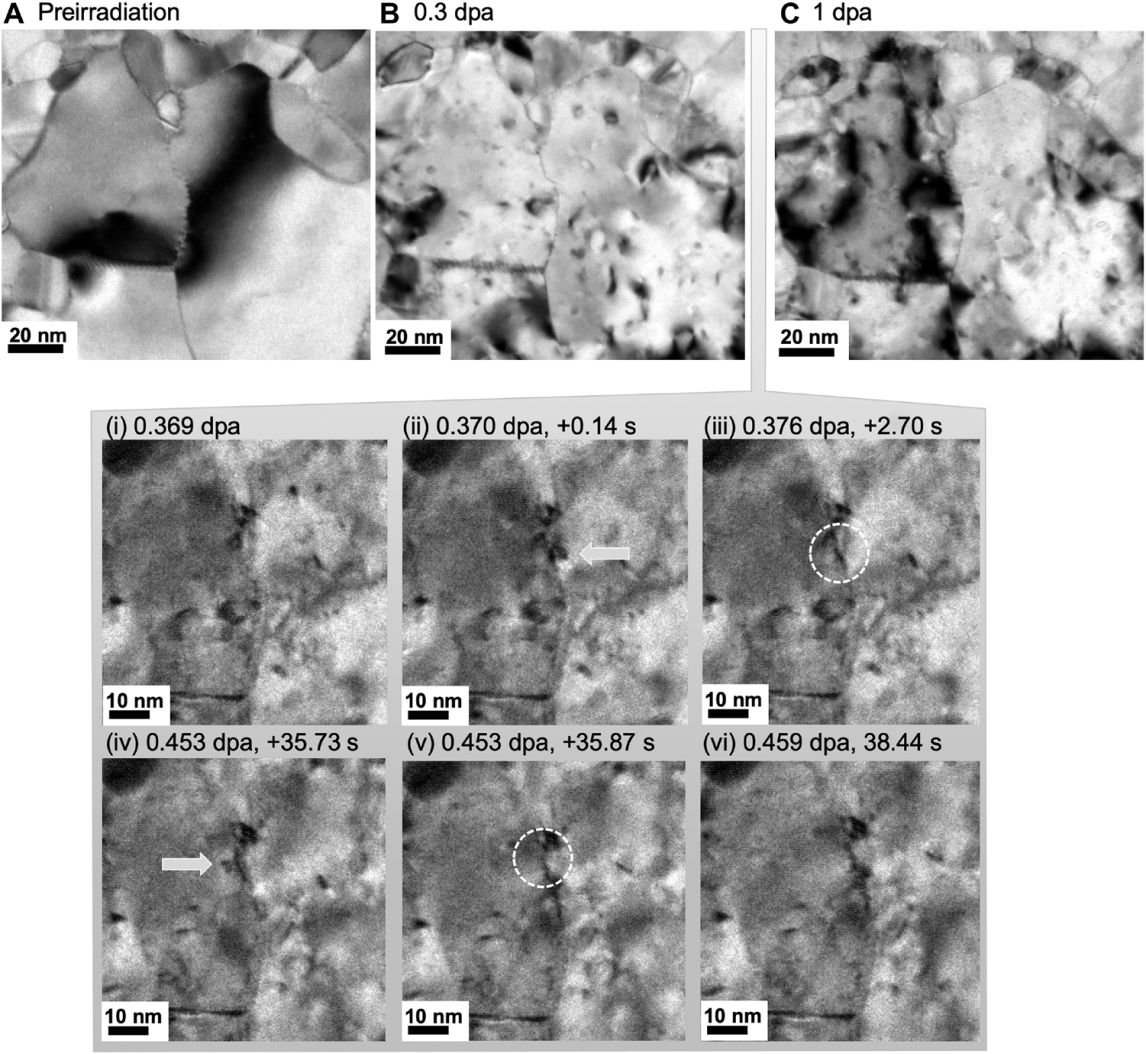
Evolution of the Σ3 GB during in situ TEM ion irradiation. (**A**) Preirradiation, (**B**) 0.3 dpa, and (**C**) 1 dpa. (i to vi) A series of still frames taken from in situ TEM. Movie S1 (0.369 to 0.459 dpa) illustrates the localized interaction between irradiation-induced defects (extrinsic to the GB) and the faceted Σ3 {112} GB.

Having characterized the initial structure of the boundary, we next examined its response to heavy ion irradiation (2.8 MeV Au^4+^) using the in situ ion irradiation TEM (I^3^TEM) at Sandia National Laboratories (see Materials and Methods for details) (*39*). Because the ion beam and electron beam in this instrument are normal to one another, imaging or video during the irradiation is conducted with the specimen tilted 30° with respect to the electron beam (60° to the ion beam). The evolution of the microstructure can be seen in movies S1 and S2. In Fig. 2, images taken at normal incidence to the electron beam allow a comparison of the microstructure starting at the intermediate 0.3 displacement per atom (dpa) condition to both the pre-irradiated condition (movie S2) and ending at the final 1 dpa condition movie S1). From these images, we observe that extensive, localized defects have been generated by irradiation even at 0.3 dpa (Fig. 2B). We expect that these defects include dislocation loops and stacking fault tetrahedra (SFT) that appear as high-contrast features, typically about 5 nm in size, distributed throughout the microstructure (*7*). The defect density increases as a function of ion fluence and does not appear to be a function of distance from the Σ3{112} GB, as is discussed further in the Supplementary Materials (Part 1. In situ defect analysis). This observation is in agreement with previous work investigating radiation damage near a Σ3{112} GB in Cu (*40*). Despite the presence of radiation damage, the overall shape of the Σ3{112} GB does not appear to change substantially by 0.3 dpa, including the segments identified in the HRSTEM images seen in Fig. 1D.

In contrast, between 0.3 and 1 dpa, movie S1 offers evidence of a series of events adjacent to the GB segment of interest that triggered its migration. A 104-s image sequence associated with damage levels increasing from 0.369 to 0.613 dpa is shown in Fig. 2 (i to vi). Although these dynamic images do not provide the clarity present in the 0° tilt images, it is still possible to observe a series of contrast features that appear and disappear in succession. Two radiation damage structures resulting from individual cascade events are indicated by white arrows in Fig. 2 (ii and iv). These features occur near the GB, some within 5 nm of the GB segment or less. These are subsequently followed by contrast changes in the GB indicated by dashed circles in Fig. 2 (iii and v). This is especially evident in the sharp change in local GB contrast that occurs at 0.453 dpa within the segment of interest, which indicates a rapid evolution of the GB structure causing the net migration described previously. The evolution occurred in less than 0.14 s (as shown comparing Fig. 2, iv to v).

**Fig. 3.**
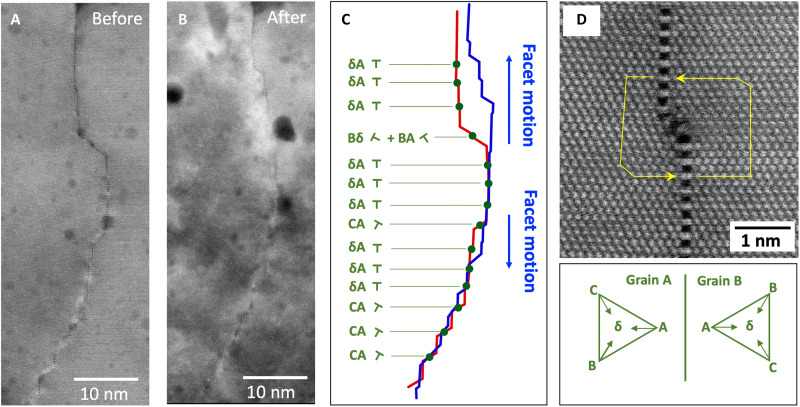
Facet junction positions before and after ion irradiation in relationship to the interfacial disconnection content measured before irradiation. (**A** and **B**) The GB facets before and after irradiation. (**C**) Plots of the facet positions measured before (red) and after (blue) irradiation. The facets have primarily moved in the upward direction relative to their initial position. The green dots on the plot for the unirradiated boundary in (C) mark the midpoints between facet junction pairs around which Burgers circuits were constructed on higher magnification images. An example of a circuit map is shown in (**D**) for a facet-junction pair with **b** = $\left(a/6)[1\bar{2}1\right]$= **δΑ**, referenced to the right crystal (grain B). The observed disconnections have Burgers vectors primarily composed of $\left(a/6)[1\bar{2}1\right]$ = **δΑ**, although other components arise where the average boundary inclination deviates substantially from $(1\bar{2}1).$

Following the in situ ion irradiation experiments, we again characterized the boundary at atomic resolution using HAADF-STEM. Figure 3 (A and B) compares HAADF-STEM images of the boundary from before and after the irradiation. Besides the changes in the GB morphology itself, the irradiation has also modified other aspects of the microstructure, including forming new cavities and enlarging the pore-like features present in the initial microstructure, as can be seen in both Figs. 2 and 3. Previous studies (*41*, *42*) have highlighted the complex interaction of pore-like features with GBs in the context of radiation damage, including the possibility of improved radiation tolerance due to the increased density of strong sinks. We expect that such pores will affect the quantitative kinetics of the defect interactions and boundary motion, but not the qualitative aspects of the mechanisms identified in this study.

To demonstrate more clearly the changes in the boundary morphology, Fig. 3C overlays plots of the boundary profiles measured from montages of atomic resolution images collected before and after the irradiation (see Materials and Methods). Following irradiation, the upper and lower side facets of the central convex feature, which protrudes into the right grain, have evidently broken into smaller segments that have migrated away from the protrusion. Overall, the predominant direction of facet motion is in the upward direction, resulting in a net motion of the GB to the right. This result is somewhat unexpected. Based solely on classical concepts of capillarity, one might have expected the protrusion to have moved leftward to reduce the total interfacial area. Although our measurements do show a small net reduction in projected boundary line length (approximately 2.7 nm over the area analyzed; see Supplementary Materials and fig. S7), the boundary motion is clearly more complicated than a simple reduction of the local curvature, suggesting more complex, longer-range interactions may also be important.

**Fig. 4.**
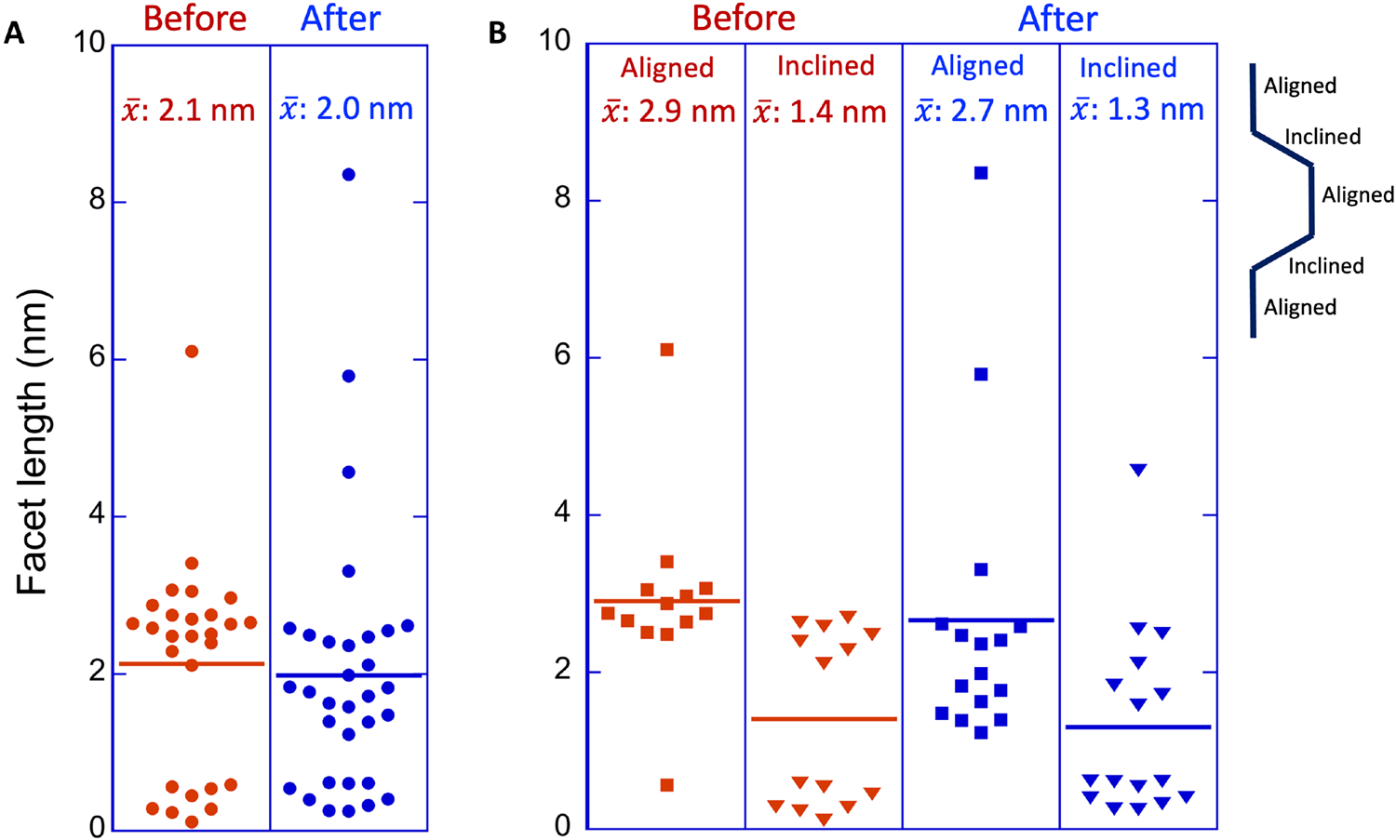
Facet length distributions measured along the length of the boundary before and after irradiation. The plots in (**A**) show all the facet lengths, whereas in (**B**), these data are partitioned into facet segments that are aligned with the general boundary direction and those that are not. While irradiation has driven an increase in population of the facets longer than 4 nm, it has also increased the population of facets in the sub–2-nm range, such that the average facet length remains roughly constant pre- and post-irradiation.

Our initial expectation from the lower-resolution video observations was that the facet motion would be accompanied by an overall coarsening of the facet length scale, as has been observed in previous studies for thermally evolving Σ3-faceted GB microstructures (*43*). To test this hypothesis, we measured the distributions of facet lengths in the pre- and post-irradiation profiles, as summarized in Fig. 4. Contrary to our initial expectation, the average facet length remains essentially constant, at about 2 nm in both the pre- and post-irradiation conditions. Because the boundary inclination is predominately aligned in the vertical direction (with reference to Fig. 3), the vertically aligned facets tend to be longer than the other facets. To ensure that this preferential alignment was not biasing our analysis, we also partitioned the facet length data to differentiate between facets that are aligned with the general boundary inclination and those that are not. Here, too, there is little change in the facet length scales following irradiation: The average length of the aligned facets is 2.9 nm pre-irradiation and 2.7 nm post-irradiation, and the average length of the inclined facets is 1.4 nm pre-irradiation and 1.3 nm post-irradiation.

Insight into this unexpected observation is provided by considering the length distributions plotted in Fig. 4. Although the total population of facets is not sufficiently extensive for robust statistical analysis, the evolution of the measured facets does present an unexpected trend. In particular, while evolution of GBs through a purely thermal route would be expected to lead overall to facet coarsening, here, the situation is more complex. Although the data show an increase in population of long facets in the 4- to 9-nm range, which tend to be aligned with the overall boundary direction, it also shows an increase in the population of short facets or steps with lengths less than 2 nm. Figure 4B shows that this increase in short-boundary segment population has occurred on both the aligned and inclined facets. The net effect of these two changes in population is that the average length scales remain constant. This observation is consistent with the notion that the irradiation is promoting the nucleation of new boundary steps, in the form of facet junction pairs and disconnections, while at the same time driving the overall boundary motion.

As noted above, before irradiation, the two grains were misoriented slightly from the exact Σ3 orientation (grain A is rotated in the clockwise direction with respect to grain B by 3.2 ± 0.1°). The measured misorientation remained unchanged at 3.2 ± 0.1° following irradiation. To better understand the relationship of this intergranular misorientation to the interfacial defect content, we analyzed the pre-irradiated boundary using circuit mapping procedures (see Materials and Methods and the Supplementary Materials). These measurements are summarized in Fig. 3C, and an example circuit is shown in Fig. 3D. The analysis identified dislocation content at every step or pair of facet junctions along the boundary. This observation suggests a linkage between the positions of the facet junctions and the positions of the disconnections present to accommodate the intergranular misorientation. The circuit analysis identified Burgers vectors primarily of type (a/6)<112> with **b** = **γΑ** (with reference to the Thompson’s tetrahedron in right grain of Fig. 3D). We also observed a few (a/2)<110>-type defects, one with **b** = **BA** in combination with a (a/6)<112>-type defect (**b** = **Bδ**) associated with the upper side facet of the larger protrusion, and several with **b** = **CA** located near the bottom of the GB.

Because the boundary is nonplanar and there are several different types of defects, establishing an exact relation between the dislocation content and misorientation is difficult. However, we can estimate the rotation associated with these defects using θ ≈ 2 sin^−1^((*b*/*L*)/2), approximating the boundary as being planar [with an inclination of $\left(1\bar{2}1\right)$] and taking *L* as the average vertical spacing of the defects (*L* = 3.55 nm) and *b* as the average Burgers vector component in the [$1\bar{2}1$] direction (*b* = −0.232 nm). This crude approximation predicts a misorientation of 3.8°, which compares reasonably with the measured misorientation of 3.2°. We also note that the defects with **b** other than **γΑ** are at locations where the GB inclination deviates strongly from the vertical $\left(1\bar{2}1\right)$ inclination, and the orientation of these defects is consistent with that needed to accommodate the misorientation along these local inclinations. In short, the distribution of defects in the pre-irradiated boundary is consistent with that required to accommodate the coherency strains that result from deviating from the exact Σ3 misorientation, and the positions of these defects are closely linked to the facet junction and step distribution.

We were unable to conduct an equivalent analysis of the distribution of dislocation content in the post-irradiated boundary because of insufficient image quality. However, the fact that the intergranular misorientation remains unchanged following irradiation implies that the net, geometrically necessary dislocation content associated with this misorientation has remained fixed. This is not to say that the ion irradiation has not introduced additional defects into the interface. Rather, any additional radiation-induced defects have not organized in a way that affects the intergranular misorientation. For instance, one would not expect point defects accumulating at the interface in the form of disconnection loops to affect the global misorientation because the loops will not contribute to the net Burgers vector density within the interface.

## DISCUSSION

The preceding observations demonstrate the complex, dynamical behavior exhibited by strongly faceted GBs subjected to ion irradiation. Previous analyses have shown how local increases in atomic jump rates, due to either the collision cascade or the associated thermal event, can enhance GB mobility, with subsequent boundary motion being driven by capillary forces to locally reduce the interfacial curvature and globally reduce the total interfacial area and free energy (*10*–*12*). Such analyses have been presented, however, in the context of general random high-angle GBs, without consideration of the effects of interfacial energy anisotropy. Moreover, recent detailed experimental observations demonstrating a lack of correlation between interfacial curvature and interfacial velocity in thermally evolving polycrystalline Ni have pointed to the inadequacy of simple curvature-based theories for predicting polycrystalline grain evolution (*18*).

One key issue is that additional considerations are needed for singular interfaces, such as the Σ3 {112} facets observed here, because they have high stiffness and lie at cusps in the energy versus inclination space (*44*, *45*). For instance, the homogeneous thermal nucleation of atomic steps, which is essential to interfacial migration, is prohibitively difficult at singular interfaces, thus requiring thermal boundary motion to occur through the heterogeneous nucleation of steps at preexisting defects or at boundary junctions (*46*). Another difference is that singular interfaces can sustain localized dislocation content (in contrast to general high-angle boundaries for which dislocations delocalize into a broad distribution of infinitesimally small Burgers vectors). In contrast to boundary motion occurring solely under thermal activation, under ion irradiation, additional mechanisms arise related both to the initial ion strike and subsequent defect interactions. In the following, we explore these considerations in the specific context of the Σ3 {112} facet motion observed here, discussing, first, the short- and mid-range interactions by which ion irradiation may provide a mechanism for step nucleation by roughening the interface and, second, the longer-range role of GB disconnections and their interactions under such an irradiation environment.

### Ion-induced instantaneous migration and longer-term roughening of facets

Although homogeneous nucleation of steps on singular interfaces is difficult by thermal activation alone, it does seem plausible that such processes could occur under irradiation, especially when individual cascade events develop in regions adjacent to the GB. To elucidate these mechanisms, we conducted atomistic simulations of relevant ion irradiation interaction processes and their role in producing localized morphological changes in the boundary structure. As a starting point, we constructed an interface composed of 120°-related Σ3{112} facets, each starting in their pristine state with no internal defects but joined by requisite facet junctions. We conducted different types of simulations to decouple instantaneous short-range irradiation effects, such as those due to ballistic knock-on displacement or local heating and atomic mixing due to the thermal event, from mid-range point defect interactions due to defect production from consecutive ion strikes. The simulations included consecutive ion strike/primary knock-on atom (PKA) simulations, simulations of isolated thermal events via local heating, and simulations using the Frenkel pair accumulation (FPA) model (*47*, *48*) to isolate the effect of point defect damage. Specific details regarding the different types of simulations are provided in Materials and Methods.

**Fig. 5.**
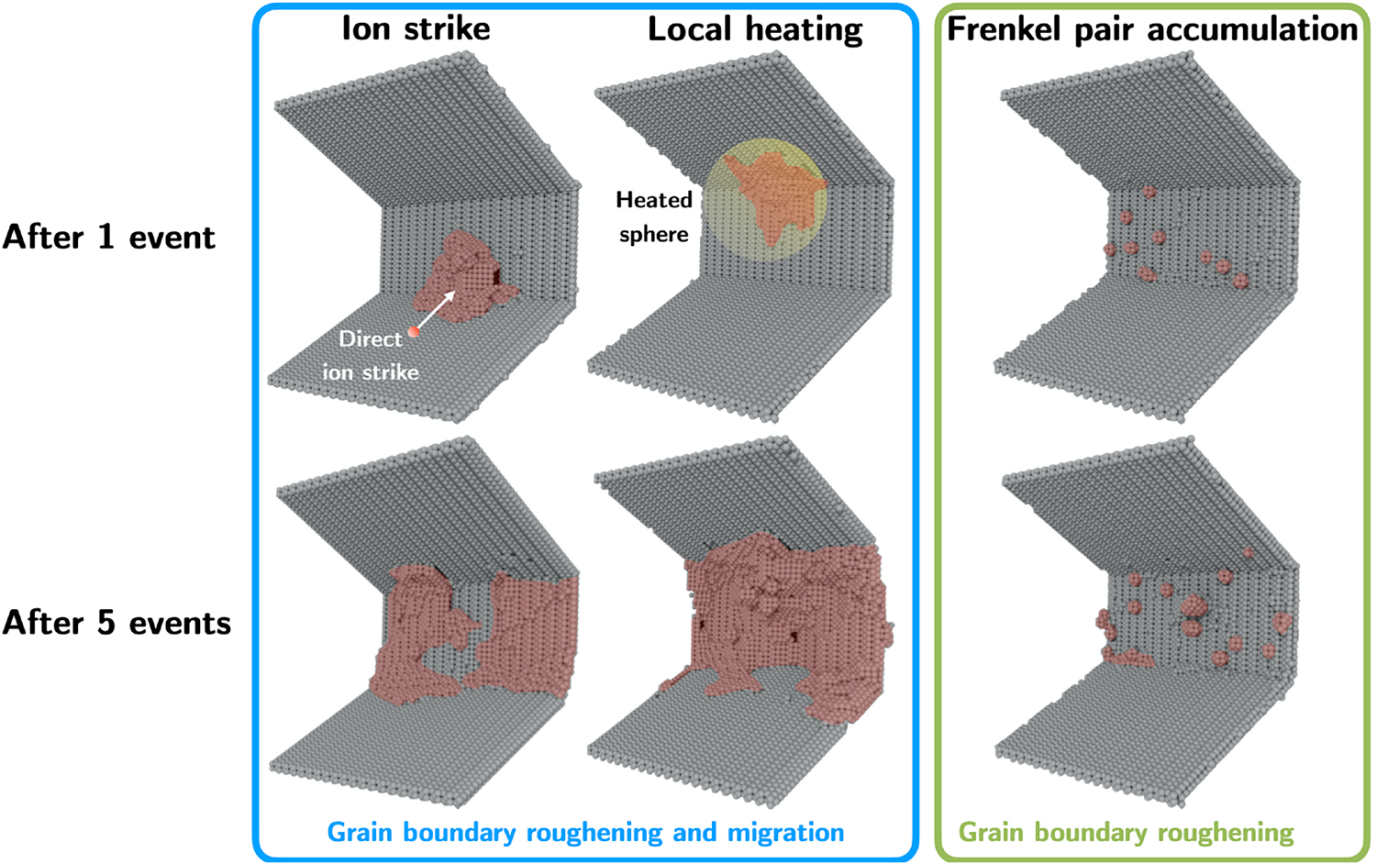
Partitioning of local mechanisms governed by ion interactions. Direct ion strike and local spherical–heated region lead to both roughening and local migration of the GB and facet junction, while FPA generates roughening of the facet junction and the Σ3{112} boundary. Top and bottom rows correspond to the postrecovery stage for each mechanism after one and five radiation events, respectively.

**Fig. 6.**
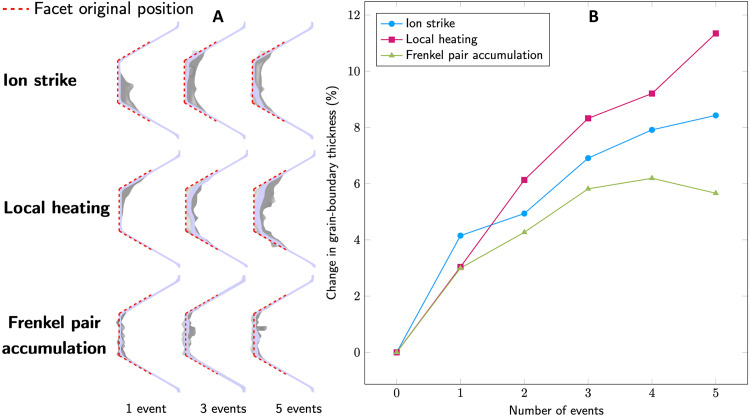
Evolution of the GB thickness due to different types of ion-GB interactions. (**A**) Snapshots corresponding to cross sections of the post-recovery stage for direct ion strike, local spherical–heated region, and FPA as a function of the number of events. (**B**) Evolution of the GB thickness as a function of the number of events for each process interacting with the facet junction and the Σ3{112} boundary.

We focused specifically on two mechanisms occurring at different time scales that could result in a change of the structure of the faceted GB, namely, (i) the instantaneous roughening and local migration of the boundary due to short-range atomic shuffling occurring in the vicinity of the facet, and (ii) the long-term roughening of the boundary caused by residual point defect production followed by subsequent defect/GB interaction. The standard method to model radiation events, the PKA method (see Materials and Methods for details), accounts for both defect mechanisms. In contrast to the ion strike and local heating models, the FPA can account for the mid-range damage of an ion strike event. In the absence of any direct contact between the thermal event and the GB, the inserted Frenkel pairs also represent the mid-range defect diffusion from ion strike events. In our PKA simulations, an individual Pt atom was assigned a velocity corresponding to an energy of 10 keV, which is a characteristic PKA energy to represent damage production during 2.8-MeV ion irradiation (*48*, *49*). In this case, local melting and atomic shuffling occurred during the thermal event phase of the cascade evolution, in which the local lattice is heated and melted through fast collisions. Eventually, as the kinetic energy is dissipated throughout the system, the atoms within the core of the cascade resolidified into crystalline lattices, largely resulting in reshuffles of atomic positions instead of point defect production. The mid-range point defect production during the recovery phase of the cascade in the PKA simulations occurred via the production of stable point defects in the later stage of the cascade development. Hence, in our PKA simulations, after the primary knock-on, a few atoms were displaced outside the thermal event region of the cascade, which converted into interstitial defects after core solidification. We show in Fig. 5 one example of a 10-keV PKA interacting with the faceted GB. Here, the GB facet experienced substantial roughening and local migration compared to the initially smooth surface. As illustrated in Figs. 5 and 6A, this mechanism becomes more pronounced at the facet junction after multiple ion strike events. As the number of ion strike events increases, we observe a generalized shift of the facet junction compared to its original position accompanied by localized morphological changes and roughening of the boundary structure. As a measure of this roughness, we characterized the change in effective GB thickness as defined by the volume of the GB region associated with non-FCC coordinated atoms, normalized by the interfacial area (see Materials and Methods). We observe that just a few cascade events can change the GB roughness by almost 10%.

To isolate the effect of the atomic mixing caused by a knock-on event during the transient thermal event, we modeled an idealized heated sphere of equivalent total kinetic energy (see Materials and Methods for details). The comparison of localized damage against the previous ion strike (PKA) configuration is shown in both Figs. 5 and 6. Our simulations show that, when the local spherical heated region interacts with the GB facet, the atomic mixing alone can cause roughening and local migration of the facet. In the absence of point defect production, as is the case in the local heating simulations, the extended damage to the GB facet is more pronounced than that seen from the ion strike simulations. Figure 6B quantifies this difference in the overall change of the GB thickness as a function of the number of events interacting with the facet junction. This observation can be explained by the lack of additional point defect production and enhanced local thermal-annealing effects.

Next, we isolated the effect of the long-term point defect production and the interaction of these defects with the boundary by using the FPA method (see Materials and Methods for details), which generates point defects in the vicinity of the GB facet bypassing the earlier stages of the cascade development (i.e., no local heating). Here, Frenkel pair defects are introduced in increments of 50, up to 250, in the vicinity of the GB. Each insertion corresponds to the average number of point defects produced by one ion strike (see Materials and Methods for details) (*50*). Although a change in the thickness of the GB facet is visible in these FPA simulations, as illustrated in Figs. 5 and 6A, regardless of the number of consecutive events, we noted the distinct absence of the local migration features seen in the full PKA simulations or in the local heating simulations for the final states. Rather, because of the largely stochastic nature of defect production, the defect/GB interaction and absorption processes resulted primarily in localized roughening of the GB thickness and local atomic structure of the GB, with no noticeable net shift or displacement of the junction.

Shown in Fig. 6B are the resulting changes in GB thicknesses after interaction and absorption as a function of the number of events. The relative changes of GB thickness produced by the local heating simulations and FPA simulations bound those produced by explicit ion strikes. Overall, our simulation results show that the defect accumulation as seen from the FPA models is different in nature than that induced by the ion strike model and the atomic shuffling models. This partitioning between short- and mid-range effects of ion strike damage near the facet junction provides important insights on the origin of the facet motion. Our results show that when an ion strike interacts with the GB facet, the instantaneous atomic mixing due to the ion strike induces a short-range shuffling of the atomic position of the GB and local migration of the GB facet that occurs at a very short time scale. In contrast, point defects from mid-range or long-range defect diffusion from far-field ion strike events not interacting directly with the facet junction result in GB roughness (*5*).

Another important consideration is how the local roughening may affect the intrinsic mobility of the boundary. Holm and Foiles (*51*) previously demonstrated that thermally induced GB roughening is accompanied by a transition from low to high GB mobility. Similarly, we would expect that the ion-induced roughening of the facets would have a comparable effect. These considerations show the duality associated with GB facet motion: not only can ion irradiation events directly drive the boundary motion but also the additional roughening through the coalescence of generated point defects may enhance the boundary mobility by providing a local means of producing the interfacial steps necessary for migration of the facet.

Since we can expect that the irradiation flux would be uniformly distributed, the overall driving force for boundary evolution with these localized short- and mid-range operative mechanisms would be a reduction in total interfacial energy (manifested by a reduction in GB area, as with conventional thermally induced curvature driven growth). However, as we discuss next, longer-range interactions with the defect structure can potentially drive more complex behavior.

### Facet junction/GB disconnection interactions

It is also important to understand the extent to which the point defect interactions couple with longer-range interactions between extended defects to affect the overall boundary motion and facet evolution. It is known that self-interstitials and vacancies do not all recombine and that self-interstitials have substantially greater mobility than vacancies (*52*, *53*). Hence, it is likely that vacancies, more so than self-interstitials, will be trapped at nearby extended defects within the matrix. The most common of such defects in the matrix would be SFTs, especially as the dose and damage density increase. In contrast, the self-interstitials that do not recombine have a greater propensity to migrate to the free surfaces and GBs, which are present at high density in nanocrystalline metals. It should be noted that the thin foil geometry (18 nm thick), which is necessary for electron imaging transparency in our experiments, creates a large sink for both self-interstitials and vacancies compared with bulk nanocrystalline metals. While this free surface will certainly reduce the concentration of interstitials, it is likely that a subset of these interstitials still reaches the Σ3 boundary. Here, they may interact directly with existing disconnections or, as discussed above, roughen the boundary and, at longer stages, form new disconnection loops on the {112} facets.

It is also interesting to consider what impact the disconnections observed at this boundary might have on the boundary’s longer-range interactions and evolution. Because the disconnections have dislocation content, we would expect them to interact elastically with other crystalline defects, such as SFTs, forming in the vicinity of the boundaries, and with each other. The interfacial disconnections in the unirradiated boundary are distributed at a density consistent with that required to accommodate the coherency strains due to deviation from the exact Σ3 misorientation. For a fixed intergranular misorientation, any insult to this locally equilibrated dislocation arrangement, whether by motion of an individual disconnection or by the introduction of new dislocation content, must result in long-range elastic interactions toward rebalancing these forces. This consideration suggests that any motion of the disconnections must occur in a coordinated fashion.

Another important interaction to consider is whether the disconnections preferentially adhere to the facet junctions themselves. Our circuit mapping results identifying dislocation content at every step and facet junction pair is consistent with such a preferential localization. This result is also consistent with prior observations in a nanofaceted body centered cubic (BCC) Fe Σ5 boundary, which also showed a linkage between the facet junction distribution and the dislocation content to accommodate deviation from the exact Σ5 misorientation (*54*).

Because they are affixed to the boundary, any motion of the disconnections in response to such elastic interactions cannot occur independently of the boundary. The dislocation content of a disconnection also imposes constraints on how it can move. In particular, the Burgers vectors of the disconnections observed in this boundary are oriented such that any motion of the disconnections with a component parallel to the long direction of the observed boundary (i.e., in the $\left[\bar{1}01\right]$ direction and parallel with the $\left(\bar{1}2\bar{1}\right)$ facets) can only occur nonconservatively (i.e., by climb, not glide). Climb of (1/6) <112> disconnections, accommodating misorientation at Σ3 {112} facets, has been observed previously under elevated temperature conditions (*37*). In contrast, here we would expect that disconnection climb would be driven by irradiation-induced point defects, most likely due to a supersaturation of highly mobile self-interstitials. The disconnections observed in the initial, unirradiated boundary (summarized in Fig. 3) have pure-edge Burgers vectors that are predominately oriented in the negative *x* direction. Climb by absorption of interstitials would thus allow for a component of motion in the positive *y* direction (i.e., upward with respect to Fig. 3). If the disconnections remain attached to the facet junctions, then such climb will manifest in the coarse scale evolution of the facet morphology.

**Fig. 7.**
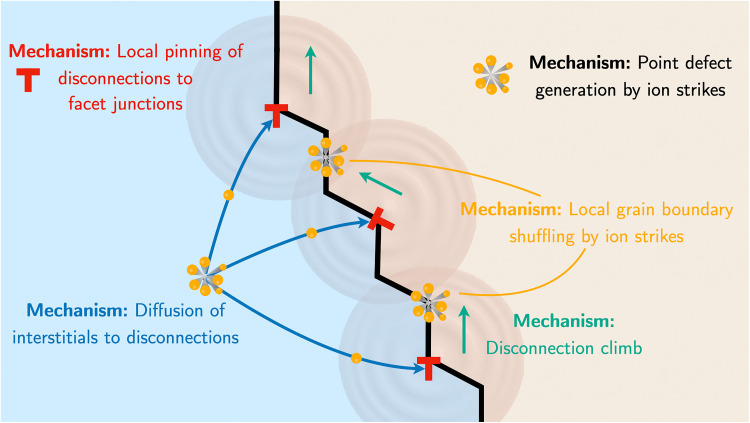
Evolution of GB facets occurs through coupling of several mechanisms at multiple length scales. Short-range effects include the restructuring of the boundary, including local step nucleation, induced by the ion irradiation. Longer-range interactions include diffusion of ion-induced point defects to the boundary where they can drive climb processes and elastic interactions between GB disconnections that, if pinned to the facet junctions, can drive coordinated motion of the facets.

In summary, our analysis suggests that the irradiation-induced evolution of GB facets, observed through in situ heavy ion irradiation, is controlled by the interplay of several mechanisms at multiple length and time scales. These mechanisms are illustrated schematically in Fig. 7. From molecular dynamics simulations, we have shown that the incident ion strikes instantaneously restructure the initially flat boundary facets. Because singular boundary facets require the nucleation of interfacial steps to migrate, such ion-induced damage and the associated thermal events provide an alternative step-nucleation pathway. Our modeling suggests that the roughening process and the associated enhanced GB mobility can be attributed to both the transient heating and the long-term accumulation of point defects at the interface occurring during irradiation. At a larger length scale, our electron microscopic observations and defect analysis have identified an array of disconnections at the interface that accommodate misorientation from the exact Σ3 orientation. Because the distribution of these disconnections is controlled by their role in accommodating the elastic coherency strains associated with the misorientation, their motion must be coupled through a long-range elastic interaction. In addition, because the observed Burgers vectors have components normal to the primary facet planes, any motion of the disconnections along the facets can only occur by climb. Assuming that the disconnections remain affixed to the facet junctions, the observations suggest that the boundary evolves nonconservatively through the diffusion of point defects, likely dominated by self-interstitials, to the disconnection cores.

Although we have focused here on the specific case of FCC Σ3{112} GB facets, we would expect these coupled mechanisms to be applicable to any GB that forms locally faceted structures and is able to sustain localized and discrete interfacial dislocation content. Hence, models seeking to incorporate interfacial energy anisotropy into predictions of irradiation-induced boundary motion should consider not only the local atomic shuffling due to the ion interactions but also the longer-range interactions associated with interfacial line defects such as facet junctions and interfacial disconnections. More broadly, a deeper understanding of the interplay between these mechanisms may suggest new strategies for tailoring interfacial structures for improved stability to irradiation-driven coarsening.

## MATERIALS AND METHODS

### Experimental

Pt thin films (18 nm thick) were deposited onto an NaCl substrate by DC magnetron sputtering (Unifilm PVD300 system) at room temperature with a base pressure of 5 × 10^−7^ torr. The deposition used a 99.99% pure Pt target and ultrahigh purity (99.999%) Ar, which was controlled at 10 millitorr throughout film growth. After deposition, the NaCl was dissolved in deionized water, and the remnant thin film was floated onto an Mo mesh TEM grid. Before irradiation, the nanocrystalline Pt was annealed in vacuum at 10^−6^ torr for 20 min at 500°C (*T*/*T*_mp_ = 0.38).

The microstructure of the annealed films was characterized before irradiation using ACOM via precession electron diffraction in a JEOL 2100 TEM with a 2.5-nm step size and 0.375° precession angle. To analyze the ACOM data, we used EDAX OIM 8.1 software, coupling the use of a single-pass grain dilation and a neighbor orientation correlation protocol with a 3° grain orientation protocol and an ambiguity resolver in Map Viewer v2. The ACOM data allowed us to identify specific GBs of interest.

The Σ3 GB was observed, both before and after the ion irradiation, using HAADF imaging in an aberration corrected HRSTEM (FEI 80-200 Titan) operated at 200 keV (FEI, Hillsboro, OR, USA). We collected a series of images from low to high magnification so that the boundary could be readily located and tracked after moving between microscopes. Circuit analyses to determine the Burgers vectors of line defects observed along the pre-irradiated boundary were conducted using the topological approaches outlined by Hirth and Pond (*55*). Examples and a discussion of the chosen reference state are provided in the Supplementary Materials (Part II. Circuit mapping of the interfacial line defects). In addition, the positions of the facet junctions were measured from atomic resolution montages of the pre- and post-irradiation boundary. To accomplish this measurement, atomic resolution images of the facet junctions (sampled at 0.0125 nm/pixel) were registered to an overview image of the entire boundary (sampled at 0.0351 nm/pixel) for both the pre- and post-irradiation conditions. These images are provided in the Supplementary Materials. The profiles were aligned to the same reference frame by aligning features common to pre- and postirradiation overview images (20 different pore-like features identified in the grains on the two sides of the boundary). Additional details concerning the image alignment are provided in the Supplementary Materials (Part III. Image alignment).

In situ ion irradiation was performed inside the I^3^TEM, a JEOL 2100 TEM integrated with a 6-MV tandem accelerator, and a broad suite of other capabilities (*39*). In this setup, the electron transparent Pt film was tilted 30° to the electron column and 60° to the incident ion source. Au^4+^ ions were accelerated through the film at an incident ion energy of 2.8 MeV to a total fluence of 4 × 10^13^ ions/cm^2^. Stopping and Range of Ions in Matter (SRIM) simulations were used to estimate the damage profile with the quick Kinchin-Pease analytical solution model and a displacement energy of 40 eV. All dpa reported here are based on this SRIM analysis. The total dose was estimated to be ≈1 dpa at a dose rate of 2 × 10^−3^ dpa/s. SRIM simulations also estimate that the total retained Au following the irradiation is approximately 5 parts per million. During the irradiation, bright-field video of the microstructural evolution was acquired at a frame rate of 15 frames/s over a field of view of ≈120 μm by 120 μm centered on the entire Σ3 GB of interest as well as the neighborhood of adjacent GBs. In addition to the tilted dynamic images provided by the video, static bright-field images were also acquired at zero tilt before and after the irradiation, as well as during one intermediate interruption at 0.3 dpa.

### Molecular dynamics simulations

**Fig. 8.**
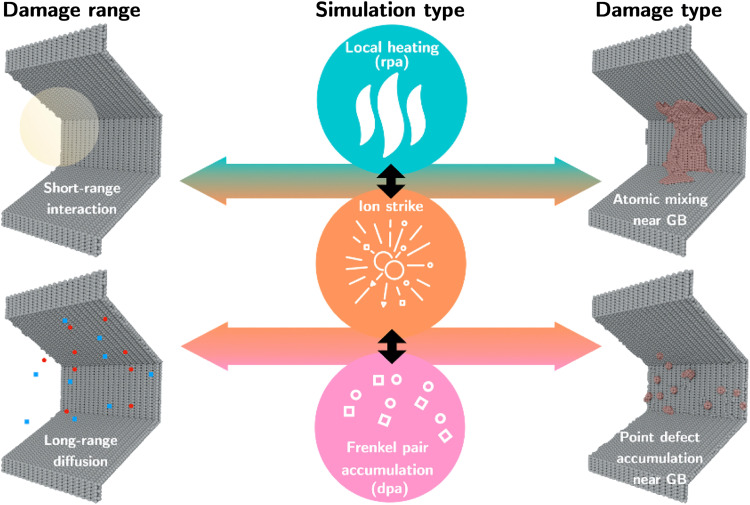
Types of simulations and resulting damage ranges and types. We used three types of simulations: direct ion strike/PKA, local spherical heating, and FPA to isolate (i) short- versus mid-range interactions of radiation events in the vicinity of the boundary system and (ii) different damage types: atomic mixing versus the accumulation of point defects near the boundary system. The direct ion strike simulations are the only ones that account for both types of damage and short- versus long-range damage.

We used the open-source large-scale atomic/molecular massively parallel simulator (LAMMPS) to perform simulations of radiation damage near the GB junctions. Pt was simulated using the embedded atom method (EAM) interatomic potential by Sheng *et al*. (*56*). This interatomic potential can accurately reproduce the phonon dispersion curves from ab initio calculations and replicate stable Σ3 GBs observed in experiments. To simulate displacement cascades, we overlaid the EAM potential with a short-range repulsive term (nuclear stopping) via the Ziegler-Biersack-Littmark (ZBL) universal screening function (*57*). The ZBL overlay provides a strong repulsive force at short range, preventing atoms from getting too close to one another during the development of the cascade. We also incorporated the effects of electronic stopping using a frictional drag force (*49*). As illustrated in Fig. 8, we performed three types of simulation to isolate damage range (short- versus mid-range damage in the vicinity of the facet junction) and damage type (atomic mixing and defect production).

### Construction of the faceted GB

**Fig. 9.**
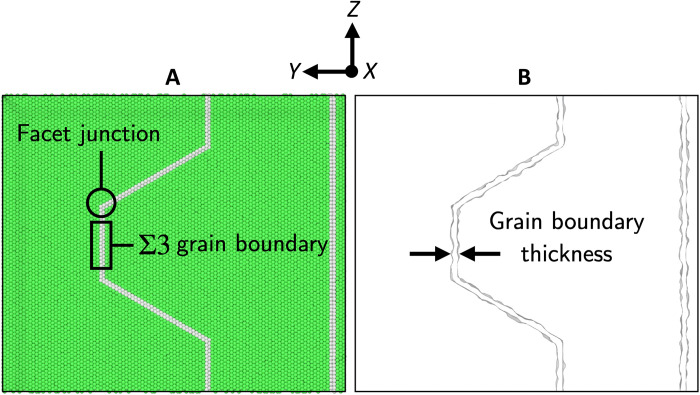
Pristine faceted Σ3 GB system. (**A**) Atomistic view of Σ3 Pt GB. Overall system is 136 Å by 257 Å by 221 Å in dimension. Atoms are colored coded by structure: green, FCC; white, disordered/GB. (**B**) Isolated primary GB volume element. Total solid volume is 144,186 Å^3^, and the total surface area is 84,426 Å^2^.

To model the observed faceted Pt GB, we constructed a three-facet GB system consisting of ideal Σ3 {112} segments with interconnecting facet junctions, as shown in Fig. 9A. Construction of the faceted GB system is similar to those of the standard bicrystal systems. Two ideal FCC bulk lattices with corresponding orientations of a flat Σ3 {112} GB were first built within the simulation cell. Matching cuts were then made in both lattices along the different {112} planes to form the multiple flat Σ3 segments. Last, two bulk lattices were brought together within 1 Å of one another to form the initial faceted GB system. The system was relaxed through an athermal energy minimization under zero pressure constraints. Subsequently, the resulting minimal energy structure was heated to 300 K and relaxed over 200 ps using a microcanonical ensemble (i.e., constant energy, constant volume ensemble, or NVE) with the Langevin thermostat and Berendsen barostat. Because of the periodic nature of the GB construction, there is a secondary flat Σ3 boundary in additional to the primary faceted boundary. However, because of the large distancing, high symmetry, and minimal lattice distortion of the Σ3 GBs, its presence can be effectively ignored.

Ion strike/PKA, local heating, and FPA simulations were conducted using this faceted Σ3 boundary geometry, using a ~500,000-atom, fully periodic system. Radiation-induced defect configurations were extracted and analyzed using a combination of adaptive common neighbor analysis (aCNA) and Voronoi volume analysis tools, as implemented in the OVITO software (*58*). The aCNA was used to identify atoms that deviated from the FCC bulk crystal structure and subsequently meshed using the Voronoi to construct a single-volume element, as illustrated in Fig. 9B. As a metric for the influence of irradiation events on the boundary structure, we characterized the effective GB thickness by normalizing this volume element by the interfacial area.

### Ion strike/PKA simulations

For the direct ion strike simulations, we initiated a collision cascade by giving a randomly selected PKA a velocity corresponding to a recoil kinetic energy of 10 keV. To allow for the cascades to interact with the GB, the initial recoil trajectory of a given cascade was randomly oriented toward the GB junction. The PKAs were initiated within 1.5 nm of the boundary. We selected this distance to ensure that the main damage region of the cascade and end of range of the cascade directly interacted with the GB junction. The displacement-cascade formation and evolution were initiated at 300 K using an NVE with a variable timestep (10^−7^ ps < Δ*t* < 10^−3^ ps) for the first 0.5 ps to accurately capture the early stage and thermal event of the cascade formation. The last 39.5 ps of the simulation used a fixed time step of 1 fs, allowing for the recovery and annealing processes of the cascade evolution to occur. Atoms in the outermost layer of the simulation box, i.e., within 1 nm of the periodic boundaries of the simulation cell, were coupled with a Langevin thermostat to absorb the cascade energy and maintain the temperature of the entire system at 300 K, mimicking the dissipation that would occur within an infinite medium. At the GB vicinity where defects were inserted, the approximate damage rate was 2.3 × 10^−4^ dpa/ps. This dose rate is much higher than the experimental setup; however, a comparison of the characteristic distance from dose rate and self-diffusion of vacancies in Pt showed that point defects do not annihilate by thermal diffusion at 300 K within the given time frame. This methodology has been used successfully in prior studies of radiation damage in atomistic systems (*49*, *59*). This process was repeated to simulate the accumulation of up to five cascade events to replicate damage build-up near the facet junction and GB system. Note that the radiation damage generated from PKA simulations account for both short- and mid-range interactions and implicitly produce both atomic mixing (during the thermal event) and point defect generation (during the recovery phase of the cascade evolution).

### FPA simulations

We used the FPA method (*47*, *48*) to simulate the isolated effect of the accumulation of radiation-induced point defects for damage levels (damage measured in dpa) without the effect of the thermal event developing in classical PKA simulations. In the FPA method, we introduced radiation damage directly as Frenkel pairs as observed at the end of a collision event in PKA simulations, bypassing the various stages of the cascade evolution. In this method, each Frenkel pair was generated by randomly displacing an atom from its original lattice position to a distance beyond instantaneous recovery (0.5 to 1.5 nm). At each time interval, we introduced *N* = 50 random Frenkel pairs, followed by a 40-ps relaxation of the entire simulation cell. The number of Frenkel pairs is chosen to match the number of dpa produced by a single 10-keV PKA. Depending on the model used, the Norgett-Robinson-Torrens (NRT-dpa) model or the athermal recombination corrected dpa (arc-dpa) model (*60*), *N* ranges between 10 and 100 Frenkel pairs. To match the number of consecutive ion strike events, the FPA insertion process was repeated five times (i.e., *N*_total_ = 250). The rate at which Frenkel pairs are inserted corresponds to the same dose rate simulated in the ion strike simulations.

### Local heating simulations

We replicated the effect of atomic mixing induced by the thermal events of cascades by using a local heated sphere interacting with the facet junction and GB system. Here, in lieu of modeling energy transfer through numerous atom-atom collisions, the total excess kinetic energy was instead instantaneously redistributed to a spherical group of atoms. The energy redistribution process at the atomic scale was conducted through temperature rescaling using the correlation $T=\frac{2}{3k}\mathrm{KE}$, where *k* is the Boltzmann constant. The temperature was rescaled up from the average ambient of 300 K to redistribute a 10-keV event such that $T_{\text{heat}}=\frac{2}{3k}[\frac{3}{2}k(300)+\frac{10,000}{N}],N\sim 2000$. This number of atoms *N* is estimated as the number of replaced atoms during the thermal event phase following the estimator proposed by Nordlund *et al*. (*60*) for a 10-keV cascade in Pt. Systems with a heated spherical volume were relaxed in an NVE over 40 ps to allow melting, atomic shuffling, and resolidification. This procedure was repeated five times to simulate the effect of atomic mixing after five consecutive events. Here again, the rate at which local heated spheres are inserted corresponds to the same dose rate simulated in the ion strike simulations.
