# Supplementary material for: Irradiation-induced grain boundary facet motion: In situ observations and atomic-scale mechanisms
Source: Sci Adv. 2022 Jun 10;8(23):eabn0900. doi: 10.1126/sciadv.abn0900 (PMC12419129; doi:10.1126/sciadv.abn0900)
Supplement: Supplementary file 1 — Supplementary Text Figs. S1 to S7 Tables S1 and S2 [file sciadv.abn0900_sm.pdf]

Science Advances
AAAS

Supplementary Materials for

Irradiation-induced grain boundary facet motion: In situ observations and atomic-scale mechanisms

Christopher M. Barr *et al.*

Corresponding author: Douglas L. Medlin, dlmedli@sandia.gov

Sci. Adv. **8**, eabn0900 (2022)
DOI: 10.1126/sciadv.abn0900

The PDF file includes:

Supplementary Text
Figs. S1 to S7
Tables S1 and S2
Legends for movies S1 and S2
Legend for data S1

Other Supplementary Material for this manuscript includes the following:

Movies S1 and S2
Data S1

Supplementary Text

1. In-situ Defect Analysis

For the analysis of defects in the in-situ TEM videos of irradiated platinum, we employed a manual approach utilizing employing image filters within the ImageJ package of analysis software. Still frames were extracted from the videos in 28 second intervals for analysis. These images were processed with ImageJ by the application of a sequential bandpass filter, gaussian blur, integral contrast normalization, and thresholding to generate a black and white mask of potential defects as shown in Figure S1.B. While the masks generated from thresholding were well representative of the defects in the TEM image, they did not capture each defect with perfect accuracy, so a manual review was conducted to filter the mask's false positives and locate defects that were not captured by image processing. A defect was identified as either a single high-contrast black spot or a loop; those directly joined to the grain boundary were not considered in the total. Defect counting was only conducted in regions adjacent to specific grain boundaries. These regions were defined by 10 nm intervals from the grain boundary and were superimposed over the TEM image alongside the defect mask from processing as shown by Supplemental Figure 1(c).

The number of defects within regions from 0-10 nm, 10-20 nm, and 20-30 nm of the grain boundary were counted and normalized by the area of the region to calculate the defect density as a function of time and distance from the grain boundary. Both supplementary videos were analyzed with the same method, and the results from the sample of frames of each video are summarized in Figure S2.

2. Circuit mapping of the interfacial line defects

The dislocation content associated with the interfacial line defects observed along the boundary was analyzed using circuit mapping procedures as developed by Hirth and Pond (*55*). In this approach, a circuit loop is constructed around the interfacial defect, passing through both crystals. This loop consists of two separate paths in the two adjacent crystals. These paths start and end at the interface on the two sides of the defect and the steps along the paths are composed of perfect lattice translation vectors. In general, the path must also include the interface crossing vectors, which will depend on the local interfacial relaxations and thus are not topological. However, if the interface crossings can be chosen at crystallographically equivalent locations on terraces or facets with parallel inclination, then these interface crossing vectors cancel, and the circuit can be analyzed entirely in terms of the two paths of perfect lattice translation vectors. In this case, the Burgers vector of the defect is given by:

$$\boldsymbol{b} = -(\boldsymbol{C}_{\lambda} + \mathbf{P}\boldsymbol{C}_{\mu})$$

where $\boldsymbol{C}_{\lambda}$ and $\boldsymbol{C}_{\mu}$ are the two halves of the circuit in the λ and μ crystals, respectively and **P** is a matrix that converts a vector from the μ to λ crystal coordinate frames. This expression employs the **FS**/RH sign convention (e.g., with the circuit constructed in a counterclockwise sense with the line direction defined as positive in the direction coming out of the page).

The defects observed in the boundary include both isolated disconnections and facet junctions that alternate between structurally equivalent, but differently oriented facets. To analyze the dislocation content at the facet junctions we constructed circuit paths around *pairs* of facet junctions (in one case we constructed the circuit around a grouping of 4 junctions, two of which were too closely spaced to separate effectively). (See Figure S4) This procedure allowed the interface crossings to be chosen at equivalent locations and thereby cancel. The Burgers

vector locations reported in Figure 3 of the main text correspond to the mid-points between the two terminating facet junctions of each pair.

Figure S3 defines the coordinate system employed for the circuit analysis. To facilitate the discussion, we have also illustrated the orientation of the standard Thompson's tetrahedron in the two crystal orientations. A vector expressed in the crystal coordinates of the left crystal (μ) can be re-expressed into the crystal coordinates of the right crystal (λ) by multiplying the vector by the following matrix:

$$\mathbf{P} = \frac{1}{3}\begin{pmatrix} -1 & 2 & 2 \\ 2 & -1 & 2 \\ 2 & 2 & -1 \end{pmatrix}$$

For the geometry defined in Figure S3, any *perfect* crystal translation vector (1/2)<110> with an in-plane component of $(1/6) < 112 >$ (i.e., ±**Aδ**, ±**Bδ,** or ±**Cδ**) also must have out-of-plane component of ± $(1/3)[111]$ (i.e., ±**δD**). Since the HRSTEM images are a projection of the structure along the [111] direction it is possible only to determine any out-of-plane component, $\Delta z,$ for the circuit vectors or the Burgers vectors by inference from crystallography and then only to within $\Delta z\ modulo(\ |[111]|)$. To handle this ambiguity, any out-of-plane component corresponding to an integer multiple of [111] was set to zero. This assumption seems physically reasonable since a screw component of $b = a\sqrt{3}$ would be extremely large and would likely have produced large distortions in the image contrast around the defects which were not apparent in the data.

Figures S4 (a-n) show HRSTEM images of the defects summarized in Figure 3 of the main text along with the circuits constructed to analyze these defects. The corresponding circuit vectors and resulting Burgers vectors are summarized in Table S1. We have also computed the

step heights and normal component of the Burgers vectors in Table S2. We have followed the conventions of reference (*55*) for both the sign and naming of these step heights. In particular, we distinguish between step heights for the isolated λ and μ crystals, $h(\lambda)$ and $h(\mu)$ respectively, and the "overlap step height", defined as the smaller of $|h(\lambda)|$ and $|h(\mu)|$. The step heights are presented in units of the interplanar spacing of the {224} planes, $d_{224} = a/(2\sqrt{6})$.

Before discussing the observed defects, it is useful to review briefly the defects expected for the Σ3 system, The admissible disconnections (or "perfect DSC lattice dislocations") possess Burgers vectors of type $(1/6) < 112 >$ and $(1/3) < 111 >$ (or linear combinations of these vectors including full crystal lattice dislocations such as the **b**=**CA** = $(1/2)[0\bar{1}1]$ dislocations observed in several of the circuits). For a boundary lying parallel with a singular facet inclination, such a defect introduces an atomic step separating two parallel and structurally equivalent terraces. The Burgers vector of the disconnection represents the crystallographic incompatibility that must be accommodated to ensure structural equivalency on either side of the defect once the crystals are joined. As such, the set of admissible Burgers vectors can be computed from the set of difference vectors between between all the crystallographic translation vectors, **t**(λ) and **t**(μ) in the two crystals, i.e., $\boldsymbol{b}_{ij} = \boldsymbol{t}(\lambda)_i - \mathbf{P}t(\mu)_j$ (*55*).

Disconnections with different step height combinations can have the same Burgers vector. For instance, Figure S5 illustrates several possible step height combinations for disconnections with **b**=$\frac{1}{6}[1\bar{2}1]$ on a Σ3 $(\bar{1}2\bar{1})$ facet (with reference to the λ coordinate frame defined in Figure S3). The $\frac{1}{6}[1\bar{2}1]$ disconnection in Figure S5(a) joins a step of +1 d_{224} in the μ crystal to -1 d_{224} in the λ crystal as observed for circuits (a) and (f) in tables S1 and S2. In contrast, the disconnection in Figure S5(b) joins steps of +4 d_{224} (μ) to _+2 d_{224} (λ), whereas the

disconnection in Figure S5 (c) has the opposite step configuration (i.e., -2 d_{224} (μ) and -4 d_{224} (λ)). Examples of $\frac{1}{6}[1\bar{2}1]$ disconnections with these step configurations are found in circuits (c), (e), (g), and (j) (Tables S1 and S2). Larger step heights than the three examples illustrated in Figure S5 are also possible for $\frac{1}{6}[1\bar{2}1]$ disconnections, as is observed for circuits (b), (d), (i), and (k). However, as can be seen in Table S2, the difference between h(λ) and h(μ), which defines the Burgers vector component normal to the interface remains constant at $b_n = -2d_{224}$ for each of these cases.

Moreover, where the circuit encompasses two facet junctions and fully developed facet, the step heights we have reported include the contribution from the intervening facet. For instance, this is the case in circuits (l), (m), and (n) (Figure S4 and Tables S1 and S2) for which the total Burgers vector is **b**=**CA** = $(1/2)[0\bar{1}1]$. Note that for a **CA** dislocation at a planar Σ3 $(\bar{1}2\bar{1})$ facet the smallest possible step heights would correspond to h(λ)=-3d_{224} and h(μ)=0 (if the **CA** dislocation were associated solely with the λ crystal) giving $b_n = -3d_{224}$.

3. Image Alignment

To measure the facet junction and disconnection positions, we registered atomic resolution HAADF images (sampled at 0.0125 nm/pixel) to overview images of the entire boundary (sampled at 0.0351 nm/pixel) for both the pre- and post-irradiation conditions. So that we could compare the absolute positions, we aligned the two overview images using features common to both images (Figure S5). Since many aspects of the images changed between the pre- post-irradiated conditions, we manually identified and determined the location of features that had remained invariant between the two images using ImageJ. 20 features used for this measurement are shown on Figure S5. From these measurements, we determined that the image

translation of the post-irradiated image relative to the pre-irradiated image was $\Delta x=-215.2 \pm 6.4$ pixels (-7.6 ± 0.2 nm) and $\Delta y=+229.4\pm15.7$ pixels ($+8.1\pm0.6$ nm). Here, the first number indicates the average measured translation for the 20 features and the second number denotes the standard deviation of these 20 measurements. The image coordinates are defined with the pixel coordinate origin at the upper left corner of the image, with positive x going left to right and positive y going top to bottom.

Note that in our first attempt at aligning the images, we assumed that the triple junction at the top of the images would serve as a fixed reference point. However, after attempting to align the images based on that point, we noted that other features common to the two images were systematically offset, indicating that the intersection of the boundary at the triple point had shifted.

To measure the distances between the facet junctions and disconnections on the boundary, we registered a montage of higher resolution images (sampled at 0.0125 nm/pixel) to the same junctions and disconnections identified on the overview images. For the pre-irradiation case, the analysis was based on a montage of 6 images, which each encompassed between 9 and 15 junctions or disconnections. For the post-irradiation case, the analysis was based on a montage 10 images, which each encompassed between 6 and 16 junctions or disconnections. These images and the measured feature positions are provided in Supplemental File "HRSTEM_Montage_Images.pdf",

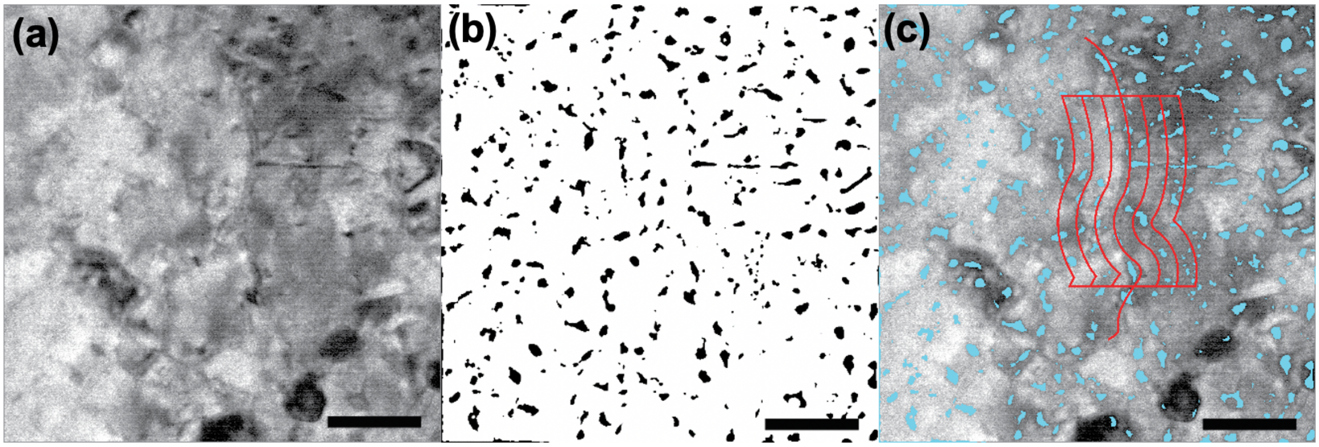

Fig. S1. The method of defect density analysis for the irradiated Pt. (a) is a raw TEM image of the grain boundary from Supplementary Video 1 and (b) is the threshold mask of defects. (c) shows of the overlay of (a) onto (b) and the outline of the grain boundary and grain boundary-adjacent regions. The scale bar is 50 nm.

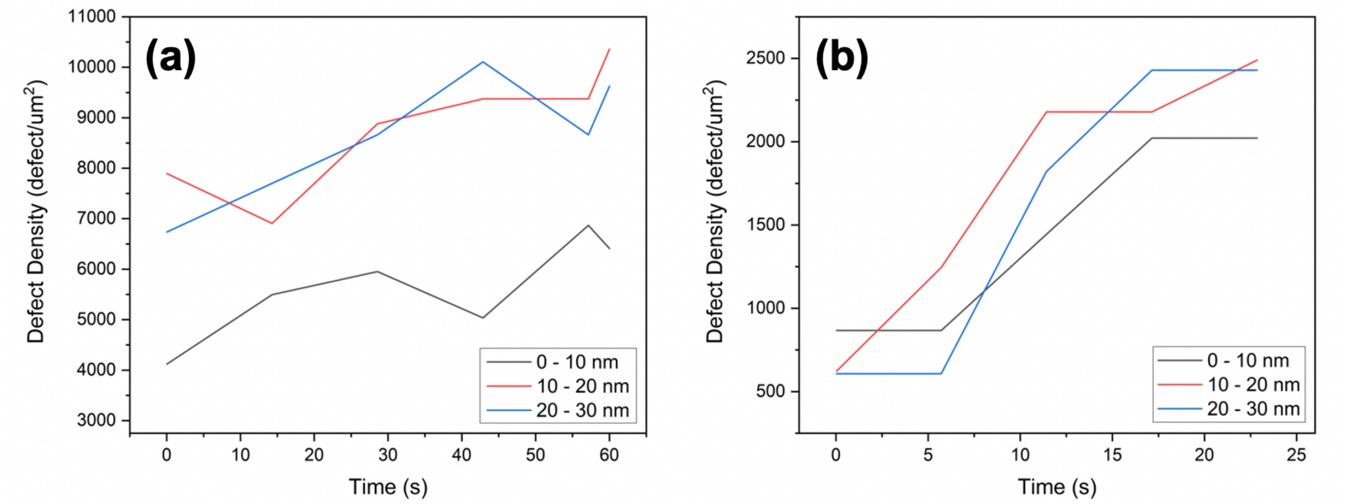

Fig. S2. Defect density in the regions adjacent to the grain boundary was determined from the video observations as a function of time during irradiation. (a) From Supplementary Video 1 and (b) Supplementary Video 2.

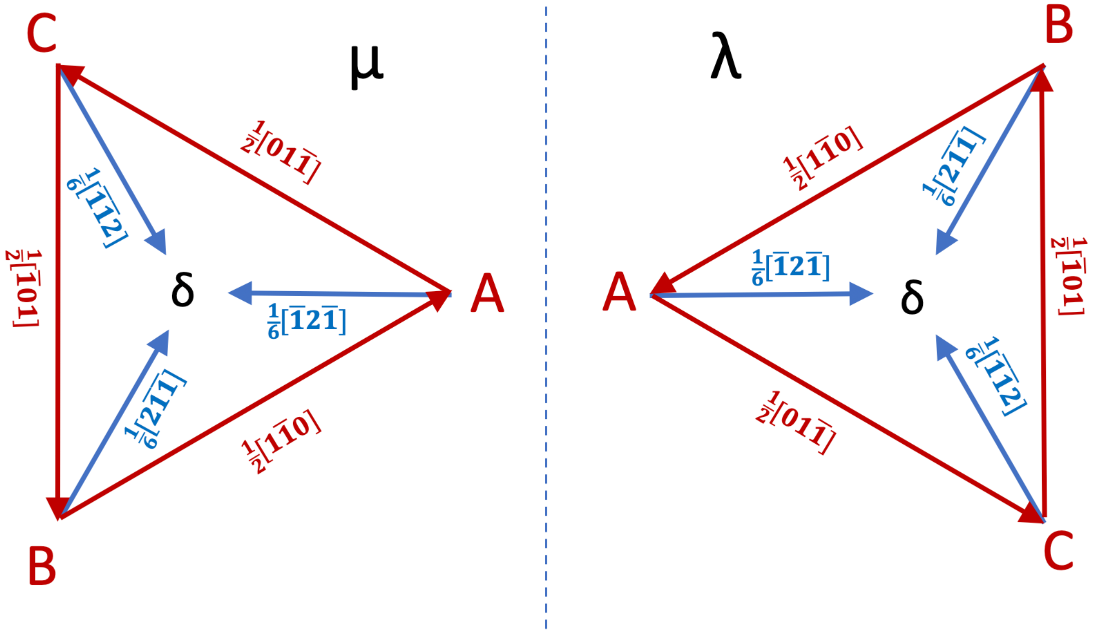

Fig. S3. Coordinate frame for the defect analysis. The directions are shown in reference to two Thompson's tetrahedra related by a 180° rotation about the [111] axis, which is oriented out of the plane. Note that *perfect* crystal translations that have in plane component (1/6)<112> (i.e., from the outer corner (A, B, or C) to δ) also have an out-of-plane component of ±(1/3)[111] (i.e., ±**δD**).

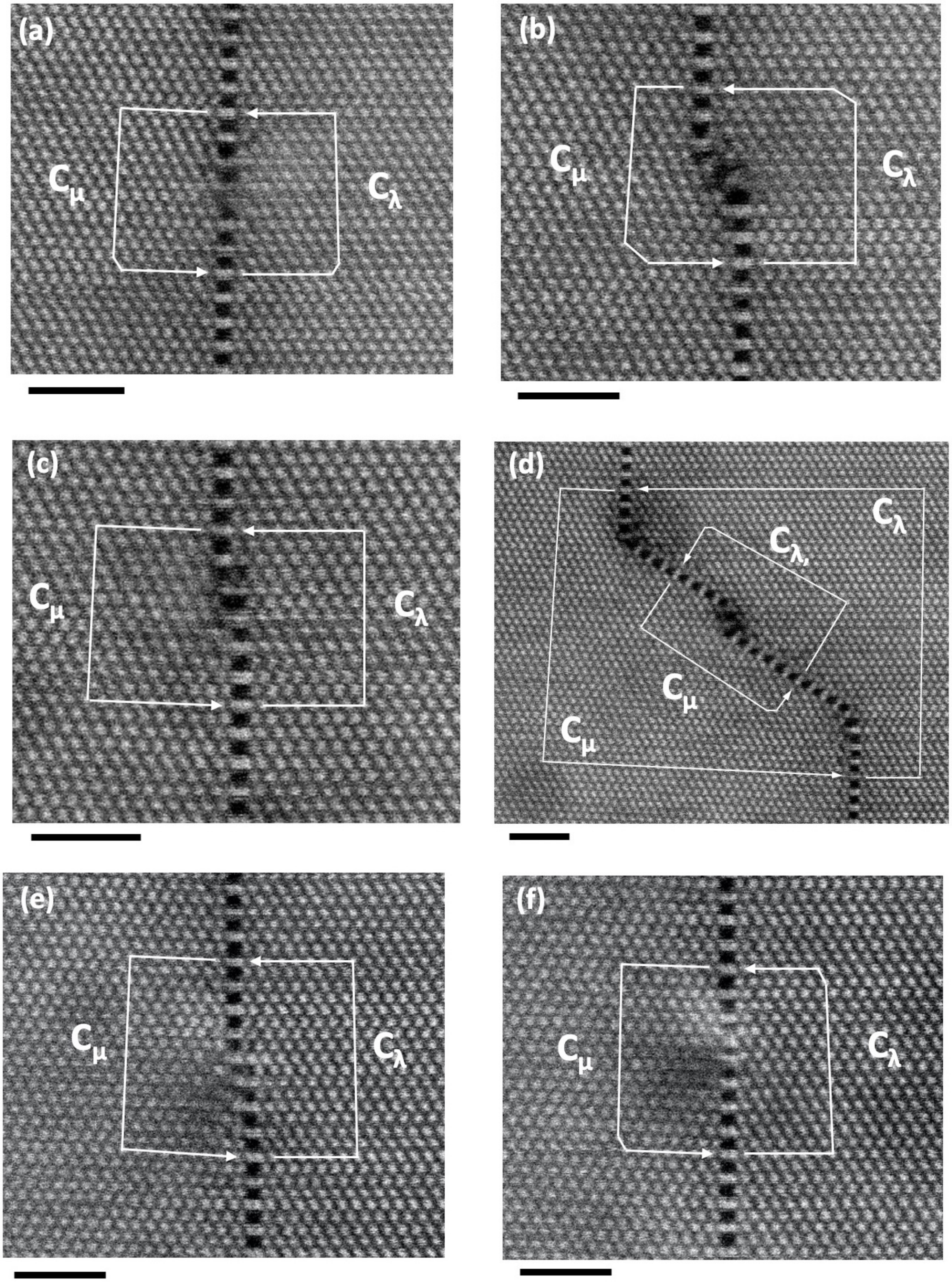

(Fig. S4. Continues next page)

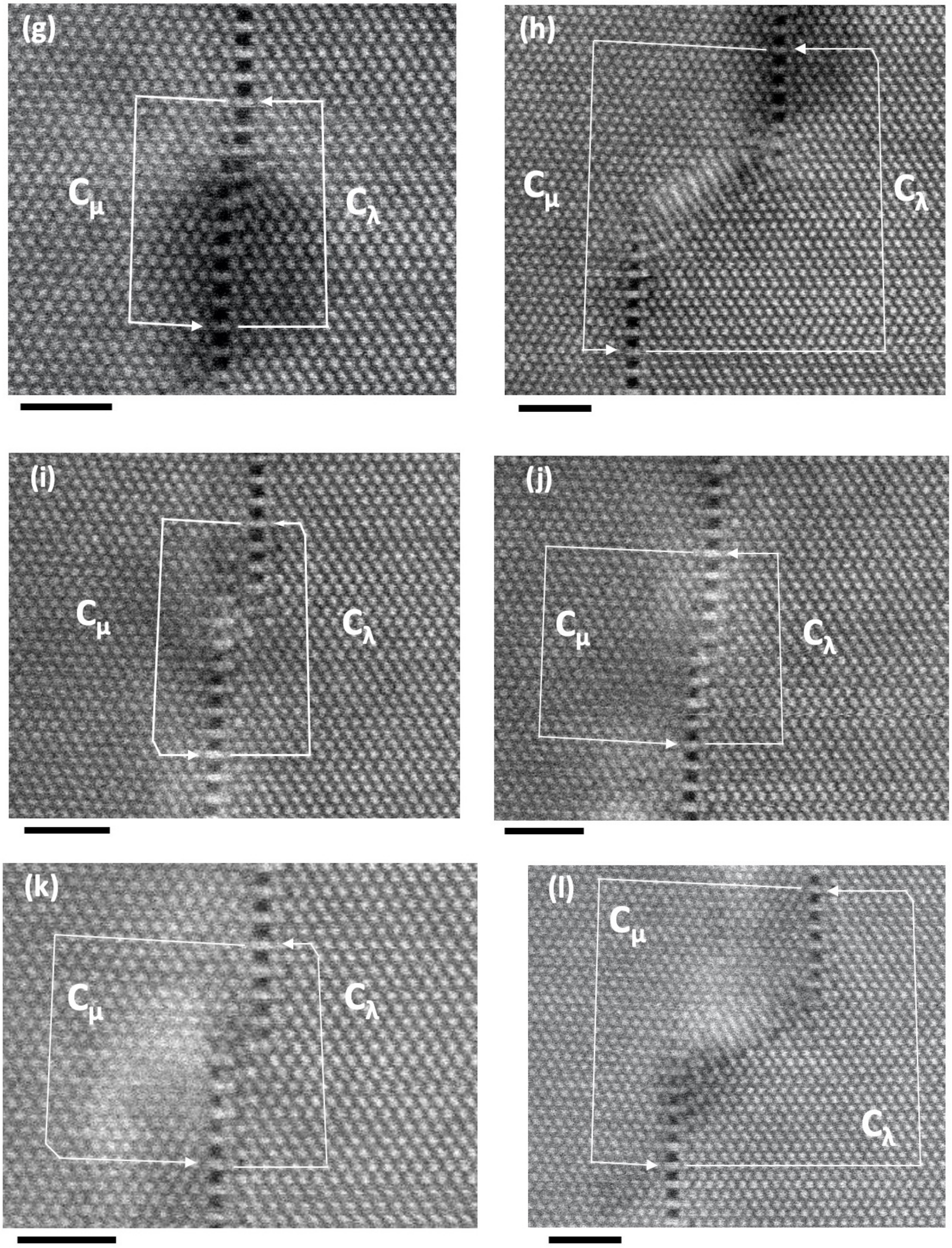

(Fig. S4. Continues next page)

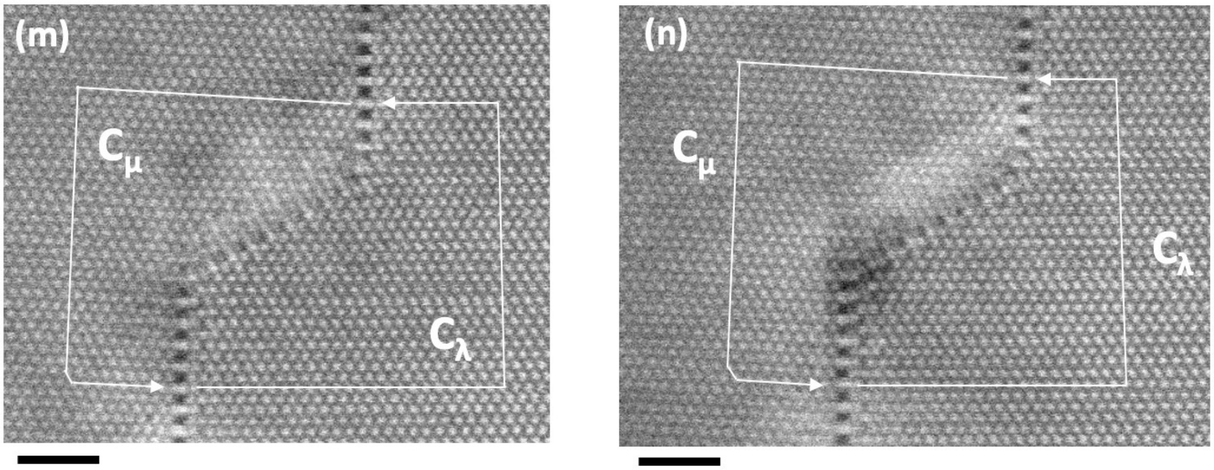

Fig. S4. The defect character of disconnections and facet junction pairs observed by HRSTEM was established by circuit mapping. These images are from the pre-irradiated Pt Σ3 boundary as summarized in Figure 3 of the main text. See Table S1 for details of the circuits and corresponding Burgers vectors. The scale bar in all images corresponds to 1 nm.

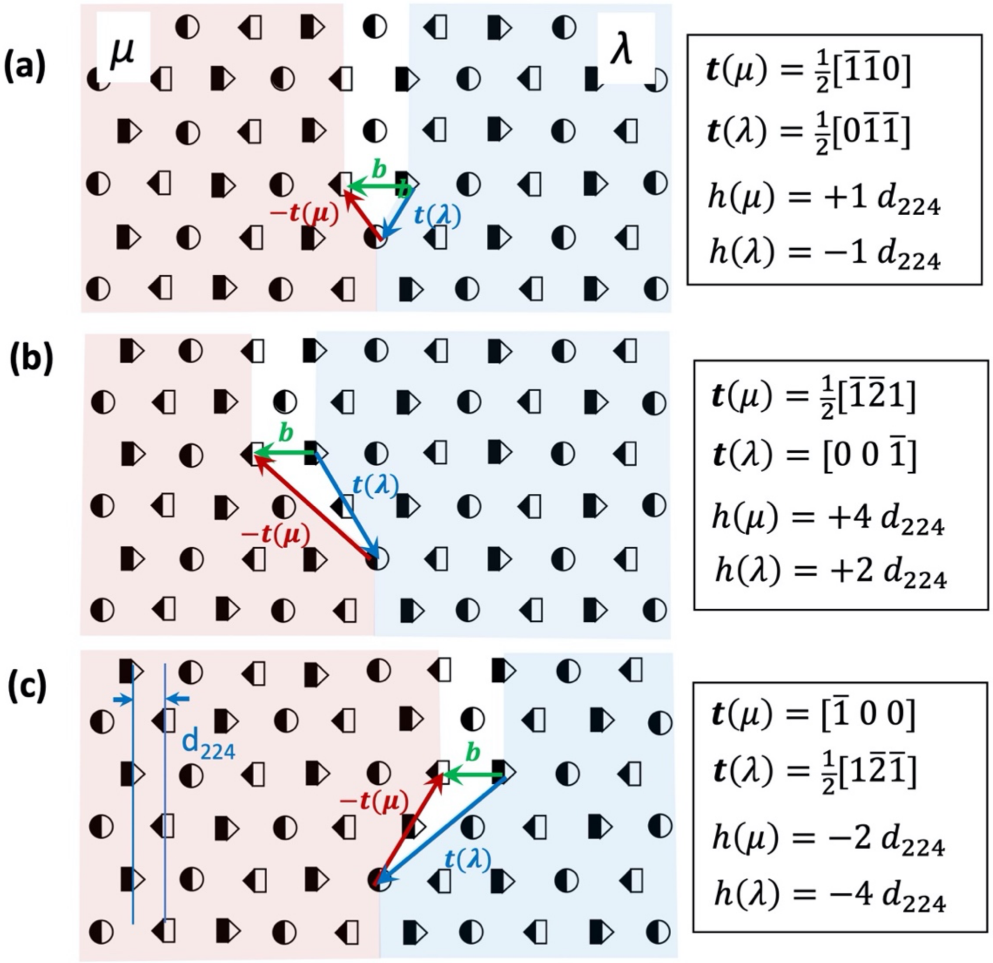

Fig. S5: The step and dislocation character of disconnections can be analyzed with reference to the dichromatic pattern. This schematic shows three different disconnections each with **b**=$\frac{1}{6}[1\bar{2}1]$, but with different step height configurations. In each case, crystallographic translation vectors, **t**(λ) and **t**(μ), terraces lying on parallel $(\bar{1}2\bar{1})$ facets. The black and white symbols represent the Σ3 dichromatic pattern projected along a [111] direction, with different shapes denoting the relative height of atoms in the projected direction. The shaded regions (pink for μ and light blue for λ) show the volumes of the crystals that would be joined at the terraces in the Volterra operation to form disconnection. The crystal coordinate system here is the same as that defined in Figure S3.

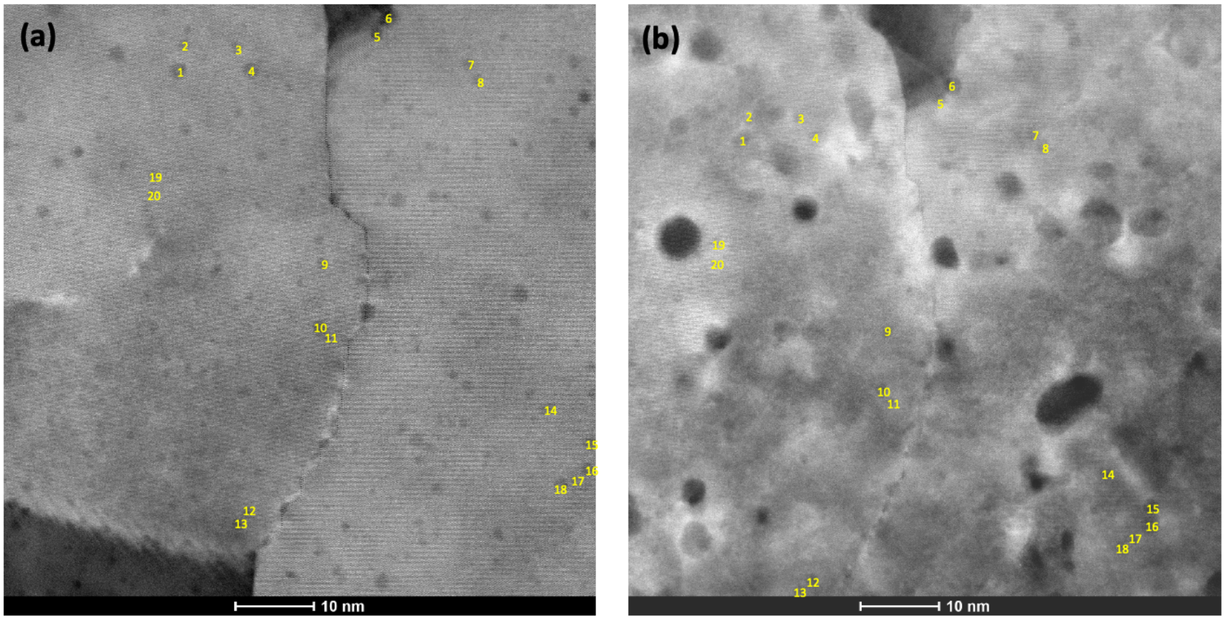

Fig. S6: The micrographs were aligned with reference to image features identified to be present both before and after irradiation. (a) prior to irradiation. (b) after irradiation.

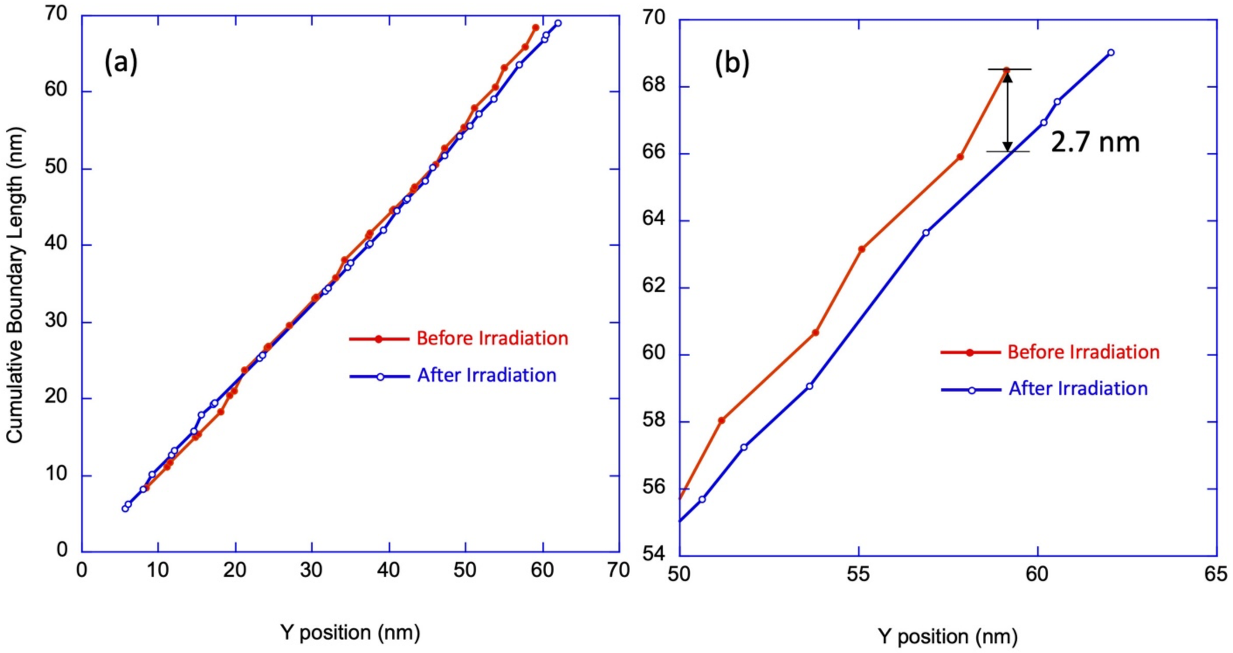

Fig. S7: Measurement of the cumulative boundary length. We measured the change in boundary length by summing the length of the boundary segments between each of the measured features before (red) and after (blue) irradiation. (a) Shows the cumulative boundary length over the full field of view. (b) Shows an enlargement of the data in (a). Integrated over the length of the observed region of boundary, there is a net reduction of approximately 2.7 nm of boundary length following irradiation.

**Circuit** (See Fig. S4)	**Circuit Vectors**	**b** (in λ reference frame)
**(a)**	$C_\lambda = \frac{1}{6}[-20,\ 1,\ 19] + \frac{1}{3}[1,1,1]$<br>$C_\mu = \frac{1}{6}[-19,-1,\ 20] + \frac{1}{3}[-1,-1,-1]$	$\frac{1}{6}[1,-2,1]$<br>**(δA)**
**(b)**	$C_\lambda = \frac{1}{6}[-17,\ -5,\ 22] + \frac{1}{3}[1,1,1]$<br>$C_\mu = \frac{1}{6}[-16,\ -10,\ 23] + \frac{1}{3}[2,2,2]$	$\frac{1}{6}[1,-2,1]$<br>**(δA)**
**(c)**	$C_\lambda = \frac{1}{6}[-17,\ -2,\ 19] + \frac{1}{3}[1,1,1]$<br>$C_\mu = \frac{1}{6}[-16,\ -4,\ 20] + \frac{1}{3}[2,2,2]$	$\frac{1}{6}[1,-2,1]$<br>**(δA)**
**(d) (outer)**	$C_\lambda = \frac{1}{6}[-31,\ -52,\ 83] + \frac{1}{3}[2,2,2]$<br>$C_\mu = \frac{1}{6}[-26,\ -56,\ 82] + \frac{1}{3}[1,1,1]$	$\frac{1}{6}[5,-4,-1]$<br>Circuit encompasses two facet junction pairs. Subtracting inner circuit leaves $\frac{1}{2}[1,-1,0]$ (**BA**)
**(d) (inner)**	$C_\lambda = \frac{1}{6}[-7,\ -28,\ 35] + \frac{1}{3}[-1,-1,-1]$<br>$C_\mu = \frac{1}{6}[-5,\ -29,\ 34] + \frac{1}{3}[1,1,1]$	$\frac{1}{6}[2,-1,-1]$<br>**(Bδ)**
**(e)**	$C_\lambda = \frac{1}{6}[-26,\ -2,\ 28] + \frac{1}{3}[1,1,1]$<br>$C_\mu = \frac{1}{6}[-25,\ -4,\ 29] + \frac{1}{3}[2,2,2]$	$\frac{1}{6}[1,-2,1]$<br>**(δA)**
**(f)**	$C_\lambda = \frac{1}{6}[-23,\ 1,\ 22] + \frac{1}{3}[1,1,1]$<br>$C_\mu = \frac{1}{6}[-22,\ -1,\ 23] + \frac{1}{3}[-1,-1,-1]$	$\frac{1}{6}[1,-2,1]$<br>**(δA)**
**(g)**	$C_\lambda = \frac{1}{6}[-29,\ 4,\ 25] + \frac{1}{3}[1,1,1]$<br>$C_\mu = \frac{1}{6}[-28,\ 2,\ 26] + \frac{1}{3}[2,2,2]$	$\frac{1}{6}[1,-2,1]$<br>**(δA)**
**(h)**	$C_\lambda = \frac{1}{6}[-60,\ 27,\ 33] + 0$<br>$C_\mu = \frac{1}{6}[-60,\ 24,\ 36] + 0$	$\frac{1}{2}[0,-1,1]$<br>**(CA)**

(Table S1 continues next page)

**(i)**	$C_\lambda = \frac{1}{6}[-35,\ 7,\ 28] + \frac{1}{3}[1,1,1]$ $C_\mu = \frac{1}{6}[-34,\ 5,\ 29] + \frac{1}{3}[-1,-1,-1]$	$\frac{1}{6}[1,-2,1]$ **(δA)**
**(j)**	$C_\lambda = \frac{1}{6}[-29,\ 4,\ 25] + \frac{1}{3}[1,1,1]$ $C_\mu = \frac{1}{6}[-28,\ 2,\ 26] + \frac{1}{3}[2,2,2]$	$\frac{1}{6}[1,-2,1]$ **(δA)**
**(k)**	$C_\lambda = \frac{1}{6}[-29,\ 7,\ 22] + \frac{1}{3}[1,1,1]$ $C_\mu = \frac{1}{6}[-28,\ 5,\ 23] + \frac{1}{3}[-1,-1,-1]$	$\frac{1}{6}[1,-2,1]$ **(δA)**
**(l)**	$C_\lambda = \frac{1}{6}[-57,\ 27,\ 30] + 0$ $C_\mu = \frac{1}{6}[-57,\ 24,\ 33] + 0$	$\frac{1}{2}[0,-1,1]$ **(CA)**
**(m)**	$C_\lambda = \frac{1}{6}[-54,\ 30,\ 24] + 0$ $C_\mu = \frac{1}{6}[-54,\ 27,\ 27] + 0$	$\frac{1}{2}[0,-1,1]$ **(CA)**
**(n)**	$C_\lambda = \frac{1}{6}[-57,\ 30,\ 27] + 0$ $C_\mu = \frac{1}{6}[-57,\ 27,\ 30] + 0$	$\frac{1}{2}[0,-1,1]$ **(CA)**

Table S1. Summary of circuit vectors and Burgers vectors for the defects presented in Figures S4 (a-n). The circuit vectors are expressed as a sum of in-plane and out-of-plane vectors in the respective λ and μ crystal coordinates defined in Figure S3. For the out-of-plane components, any integer multiples of [1 1 1] are taken as zero. Burgers vectors are expressed in the λ (right crystal) coordinate frame and defined using the **FS**/RH sign convention with the line direction defined as positive out of the page.

Circuit (See Fig. S4)	**b** (in λ reference frame)	$h(\mu)$ (d_{224})	$h(\lambda)$ (d_{224})	h (overlap) (d_{224})	b_n (d_{224})
(a)	$\frac{\mathbf{1}}{\mathbf{6}}[\mathbf{1},-\mathbf{2},\mathbf{1}]$ (**δA**)	+1	-1	0	-2
(b)	$\frac{\mathbf{1}}{\mathbf{6}}[\mathbf{1},-\mathbf{2},\mathbf{1}]$ (**δA**)	+7	+5	+5	-2
(c)	$\frac{\mathbf{1}}{\mathbf{6}}[\mathbf{1},-\mathbf{2},\mathbf{1}]$ (**δA**)	+4	+2	+2	-2
(d) (inner)	$\frac{\mathbf{1}}{\mathbf{6}}[\mathbf{2},-\mathbf{1},-\mathbf{1}]$ (**Bδ**)	-5	-7	-5	-2
		The step heights and normal component of the Burgers vector are expressed for circuit (d) relative to $\boldsymbol{n_\lambda} = [\bar{2}11]/\sqrt{6}$ and $\boldsymbol{n_\mu} = [2\bar{1}\bar{1}]/\sqrt{6}$			
(e)	$\frac{\mathbf{1}}{\mathbf{6}}[\mathbf{1},-\mathbf{2},\mathbf{1}]$ (**δA**)	+4	+2	+2	-2
(f)	$\frac{\mathbf{1}}{\mathbf{6}}[\mathbf{1},-\mathbf{2},\mathbf{1}]$ (**δA**)	+1	-1	0	-2
(g)	$\frac{\mathbf{1}}{\mathbf{6}}[\mathbf{1},-\mathbf{2},\mathbf{1}]$ (**δA**)	-2	-4	-2	-2
(h)	$\frac{\mathbf{1}}{\mathbf{2}}[\mathbf{0},-\mathbf{1},\mathbf{1}]$ (**CA**)	-24	-27	-24	-3
(i)	$\frac{\mathbf{1}}{\mathbf{6}}[\mathbf{1},-\mathbf{2},\mathbf{1}]$ (**δA**)	-5	-7	-5	-2
(j)	$\frac{\mathbf{1}}{\mathbf{6}}[\mathbf{1},-\mathbf{2},\mathbf{1}]$ (**δA**)	-2	-4	-2	-2
(k)	$\frac{\mathbf{1}}{\mathbf{6}}[\mathbf{1},-\mathbf{2},\mathbf{1}]$ (**δA**)	-5	-7	-5	-2
(l)	$\frac{\mathbf{1}}{\mathbf{2}}[\mathbf{0},-\mathbf{1},\mathbf{1}]$ (**CA**)	-24	-27	-24	-3

(Table S2 continues next page)

(m)	$\frac{\mathbf{1}}{\mathbf{2}}[\mathbf{0},-\mathbf{1},\mathbf{1}]$ (**CA**)	-27	-30	-27	-3
(n)	$\frac{\mathbf{1}}{\mathbf{2}}[\mathbf{0},-\mathbf{1},\mathbf{1}]$ (**CA**)	-27	-30	-27	-3

Table S2. Summary of Burgers vectors and Step-heights for circuits presented in Figures S4 (a-n). Burgers vectors are reproduced for convenience from Table S1. The step heights in the $\boldsymbol{\mu}$ and λ crystals, h(μ) and h(λ), respectively, the overlap step height, h, and the normal component to the Burgers vector, $\boldsymbol{b_n}$, are expressed in units of $\boldsymbol{d_{224}} = \boldsymbol{a}/(\mathbf{2}\sqrt{\mathbf{6}})$. These parameters are all computed with the interface normals defined as positive pointing into the λ crystal. Except for circuit (d), the interface normals are $\boldsymbol{n_\lambda} = [\bar{\mathbf{1}}\ \mathbf{2}\ \bar{\mathbf{1}}\ ]/\sqrt{\mathbf{6}}$ and $\boldsymbol{n_\mu} = [\mathbf{1}\ \bar{\mathbf{2}}\ \mathbf{1}\ ]/\sqrt{\mathbf{6}}$.

Movie S1 (separate file)

In-situ TEM video captured using the In-situ Ion Irradiation TEM (I^3TEM). The sample is tilted +30˚ towards to the 2.8 MeV Au^{4+} beam. The video captures data from 0.3 dpa to the final condition (1 dpa) that correlates with the still frames presented in Figure 2 of the main text. The video has been aligned to compensate for drift. (Note that the stills in the main text were rotated by 180° with respect to the video to present the data in an orientation aligned with the other images).

Movie S2 (separate file)

In-situ TEM video captured using the In-situ Ion Irradiation TEM (I^3TEM). The sample is tilted +30˚ towards to the 2.8 MeV Au^{4+} beam. The video captures data from the initial state (0 dpa) to 0.3 dpa. The video has been aligned to compensate for drift.

Data S1

High Resolution STEM (HAADF) images collected along the length of the boundary for both the pre- and post-irradiated boundaries. The file shows the positions of the images with respect to the reference overview image of the full boundary. The defect features and their measured positions in the coordinate frame of the reference overview image are also provided.
