# Supplementary material for: Irradiation-induced grain boundary facet motion: In situ observations and atomic-scale mechanisms
Source: Sci Adv. 2022 Jun 10;8(23):eabn0900. doi: 10.1126/sciadv.abn0900 (PMC12419129; doi:10.1126/sciadv.abn0900)
Supplement: Supplementary file 3 — Data S1 [file sciadv.abn0900_data_s1.zip › sciadv.abn0900_data_s1.pdf]

HRSTEM Montage images

The following slides show the grain boundary defect feature positions identified on the montages of HAADF STEM images nm/pixel) for the pre- and post-irradiation boundaries. The approximate position of each image relative to the lower magnification reference image (upper right corner) is indicated by the yellow box. The tables give the feature positions expressed in the coordinate frame of the lower-magnification reference image.

Note that the feature labels are only intended to relate identical features within a given data set (i.e., within the pre-irradiated data set or within the post-irradiated data set). They are not intended to imply feature correspondence between the pre- and post-irradiated data sets.

The features labeled d1 and d2 in the pre-irradiated data set correspond to the defects in Figures S4(a) and S4(f), respectively. Since these defects do not produce a detectable step (or more precisely since h_μ and h_λ cancel, we did not include them in our measurements of the facet length distributions.

Prior to Irradiation

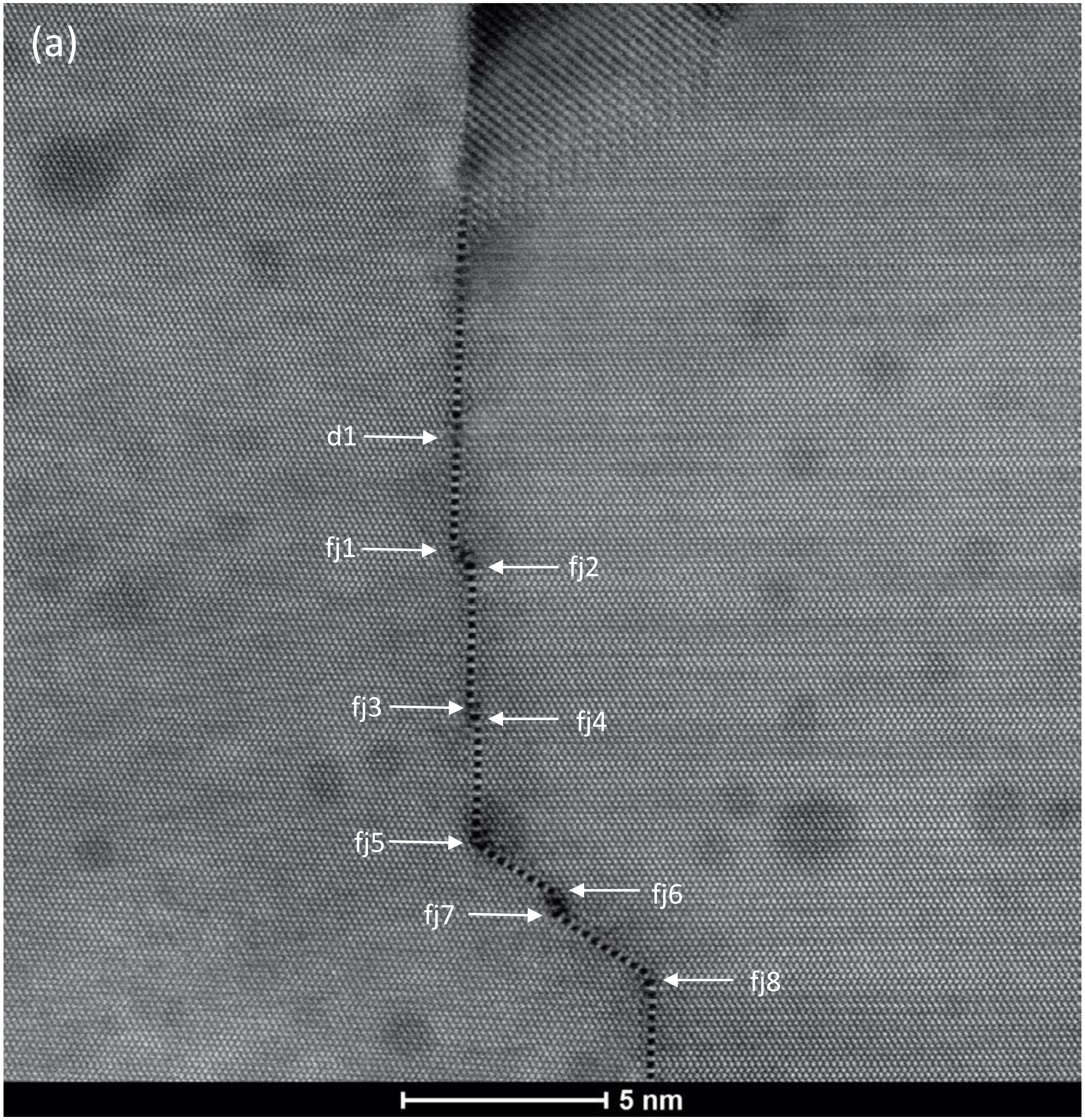

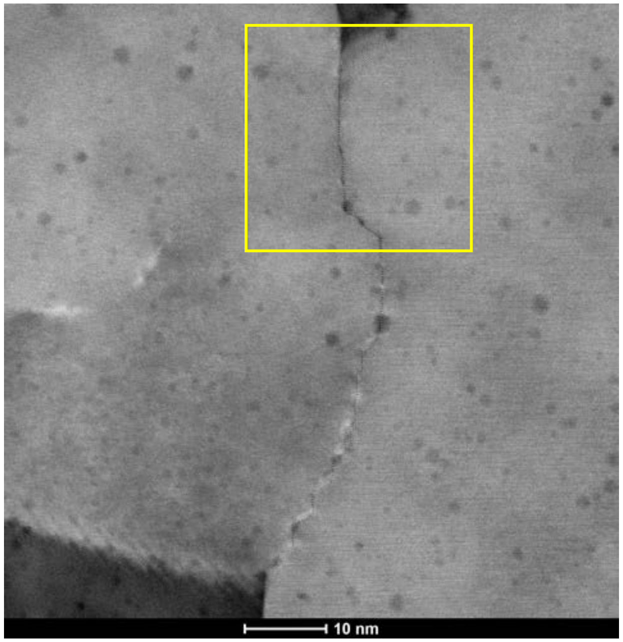

feature ID	x(nm)	y(nm)
d1	39.31	14.39
fj1	39.21	17.06
fj2	39.63	17.48
fj3	39.65	20.98
fj4	39.80	21.09
fj5	39.74	24.11
fj6	41.55	25.20
fj7	41.69	25.73
fj8	43.90	27.20

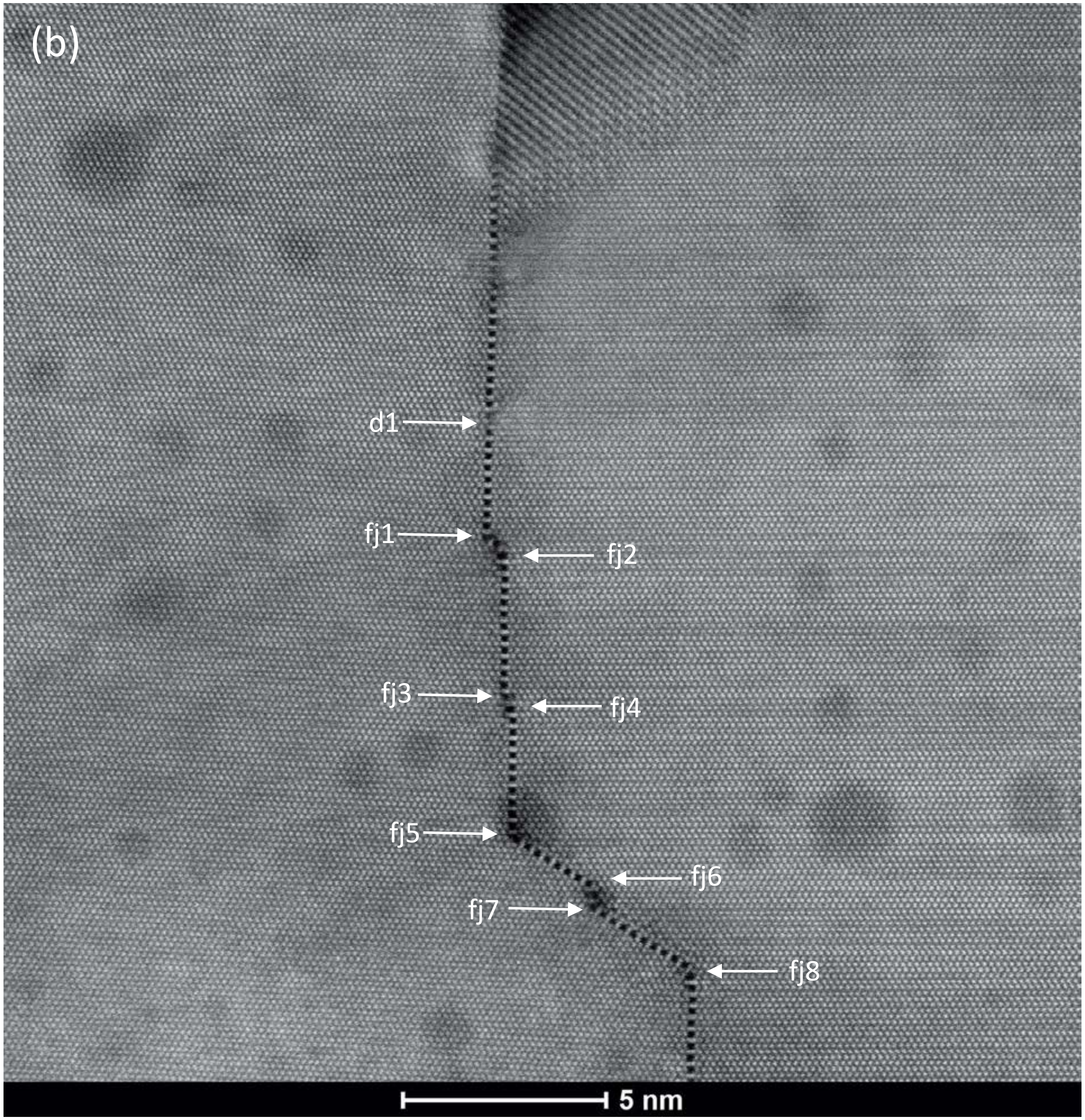

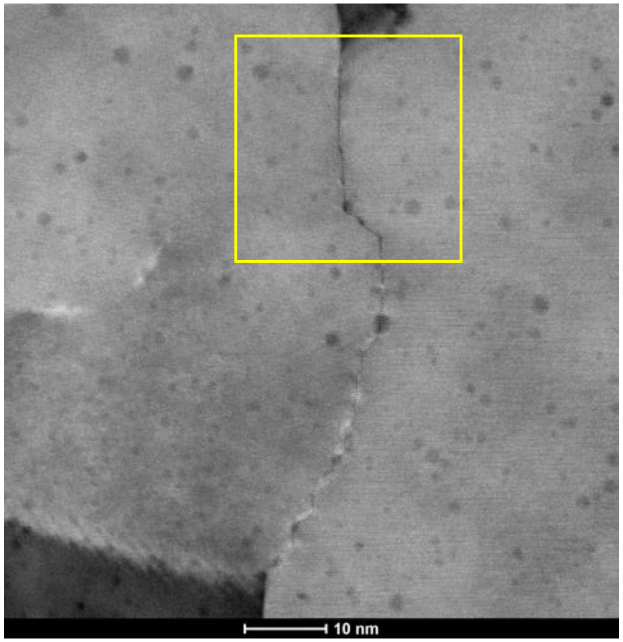

feature ID	x(nm)	y(nm)
d1	39.23	14.25
fj1	39.14	17.03
fj2	39.57	17.46
fj3	39.59	20.83
fj4	39.77	21.11
fj5	39.82	24.20
fj6	41.60	25.25
fj7	41.72	25.81
fj8	44.05	27.28

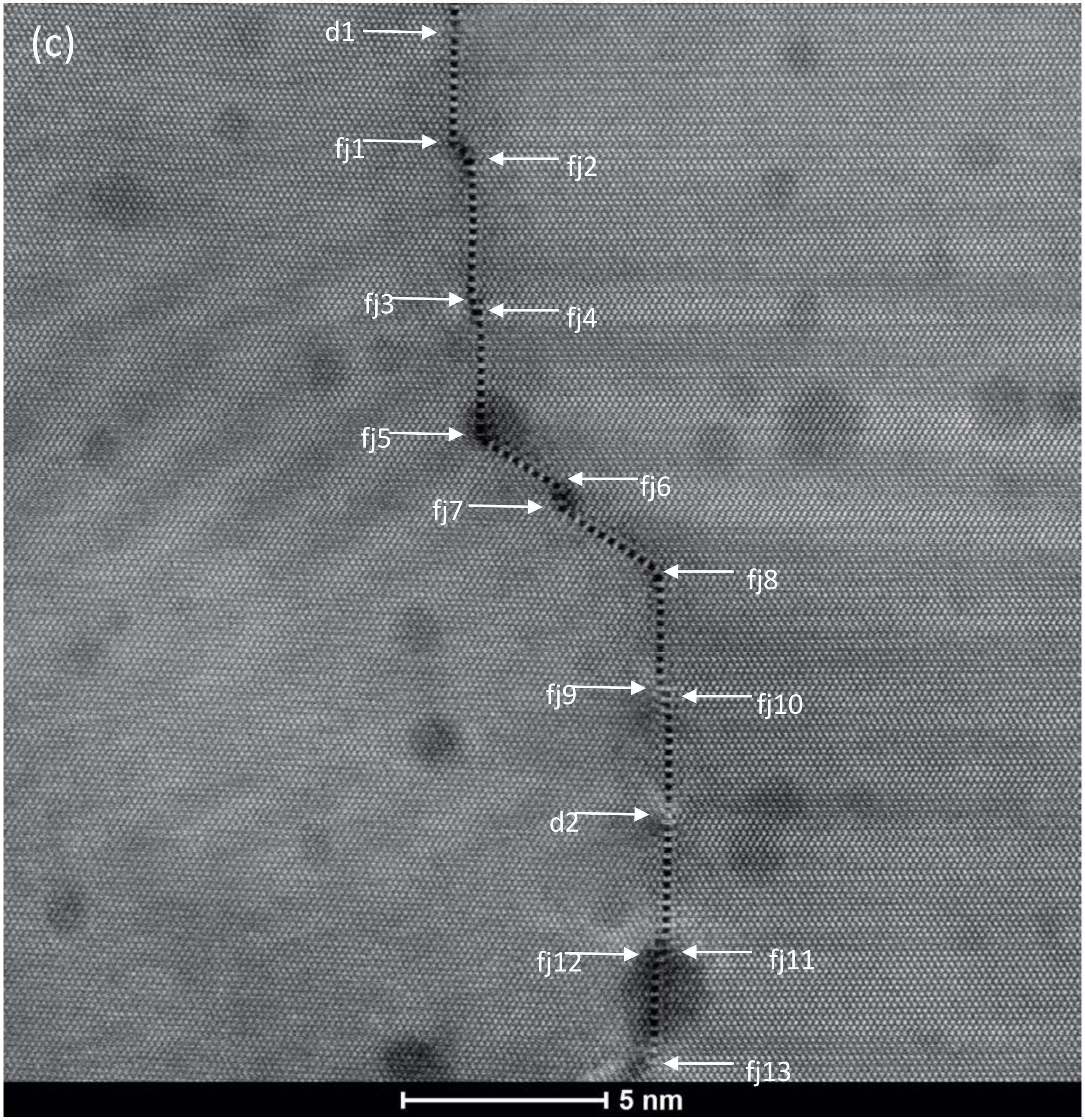

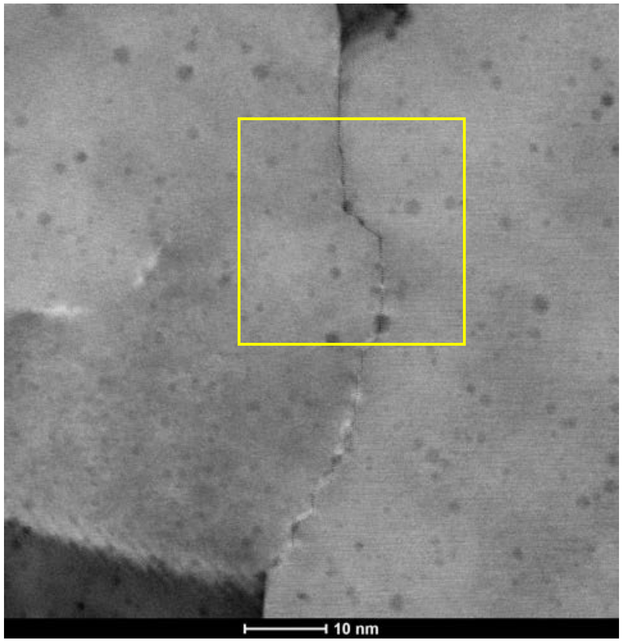

feature ID	x(nm)	y(nm)
d1	39.18	14.49
fj1	39.15	17.23
fj2	39.58	17.62
fj3	39.63	20.97
fj4	39.78	21.21
fj5	39.80	24.26
fj6	41.59	25.34
fj7	41.76	25.88
fj8	44.03	27.32
fj9	44.08	30.03
fj10	44.25	30.27
d2	44.26	33.23
fj11	44.16	36.39
fj12	43.95	36.48
fj13	43.88	38.87

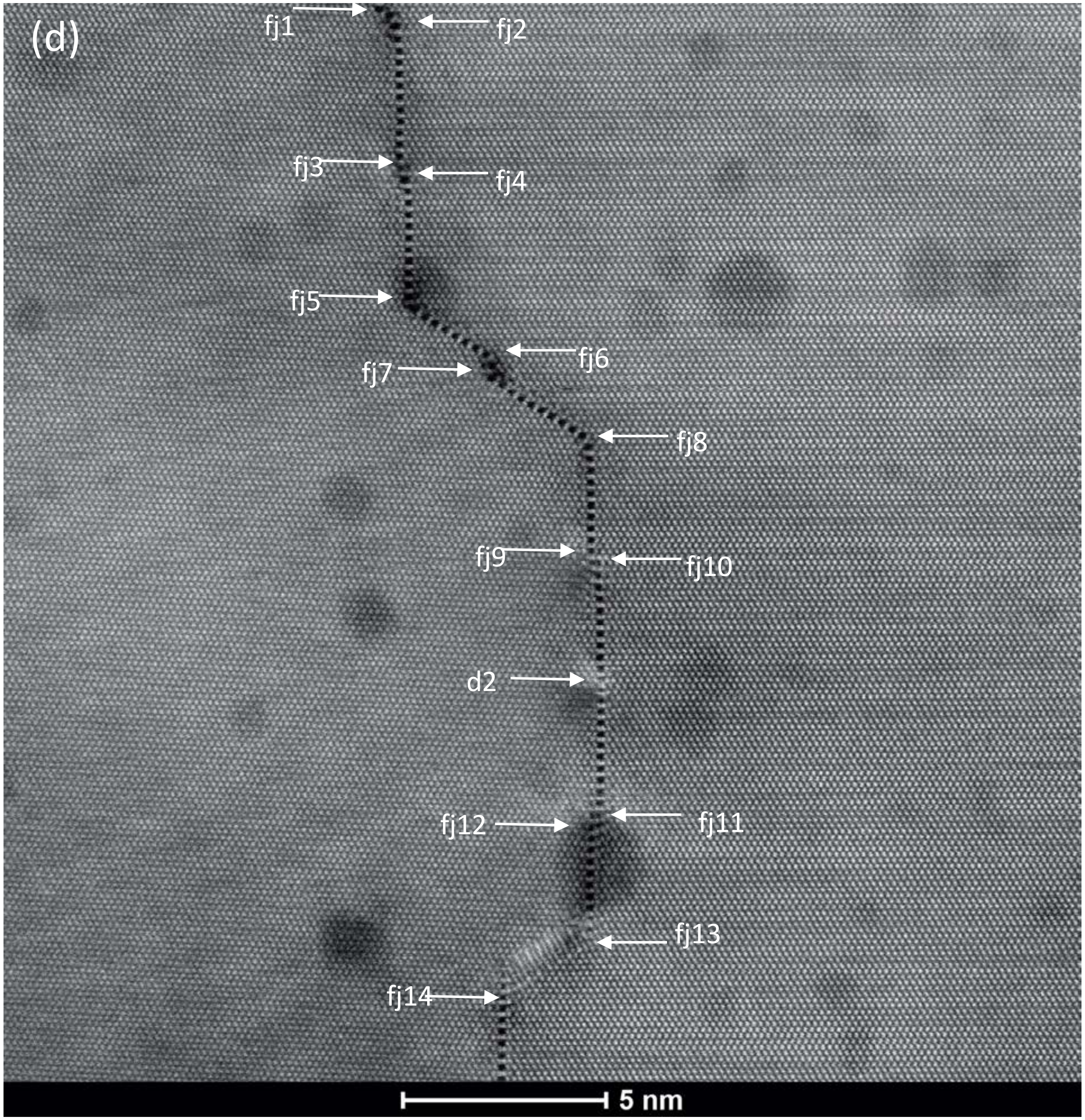

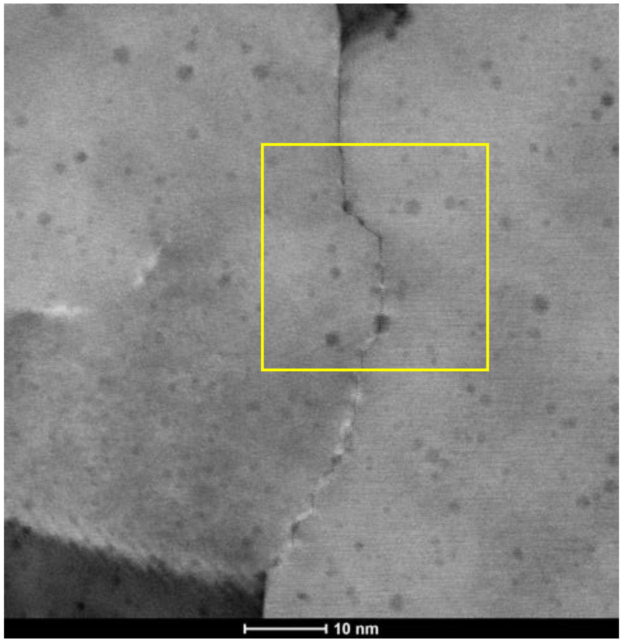

feature ID	x(nm)	y(nm)
fj1	39.03	17.11
fj2	39.48	17.49
fj3	39.55	20.90
fj4	39.68	21.19
fj5	39.76	24.21
fj6	41.60	25.34
fj7	41.75	25.88
fj8	44.01	27.34
fj9	44.10	30.28
fj10	44.23	30.51
d2	44.32	33.08
fj11	44.28	36.48
fj12	44.08	36.56
fj13	44.05	38.96
fj14	42.01	40.21

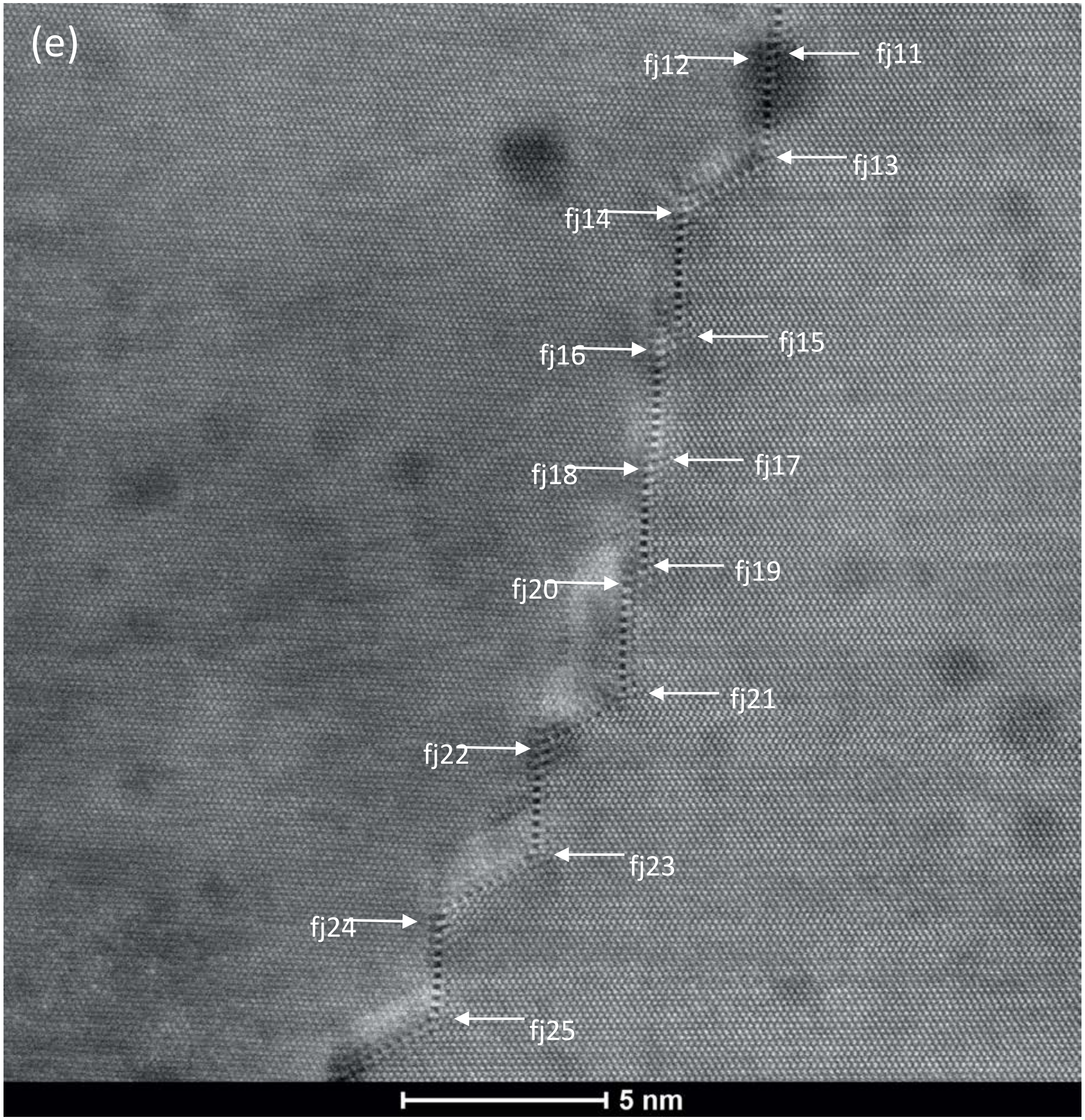

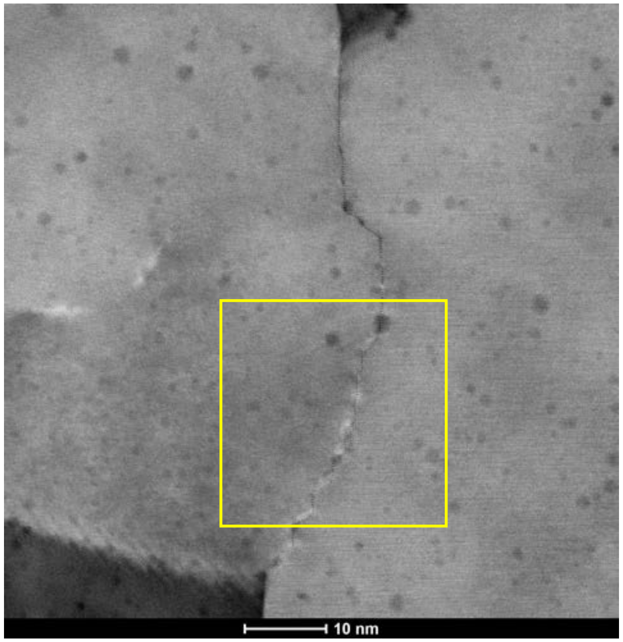

feature ID	x(nm)	y(nm)
fj11	44.25	36.62
fj12	44.00	36.69
fj13	43.95	39.34
fj14	41.91	40.54
fj15	41.83	43.44
fj16	41.40	43.58
fj17	41.28	46.54
fj18	41.06	46.65
fj19	41.03	49.09
fj20	40.61	49.38
fj21	40.50	52.24
fj22	38.51	53.22
fj23	38.45	55.75
fj24	36.18	57.23
fj25	36.03	59.90

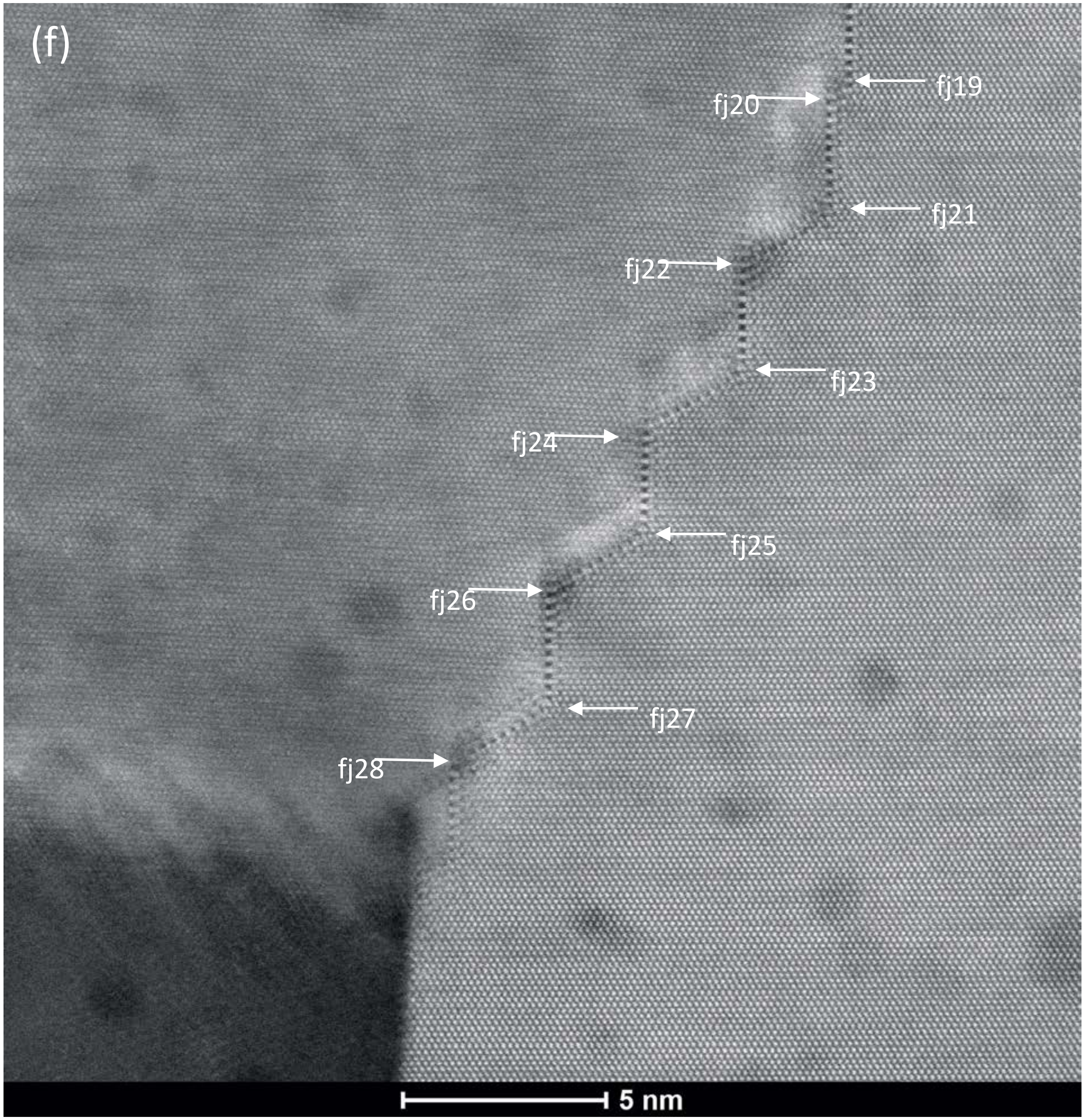

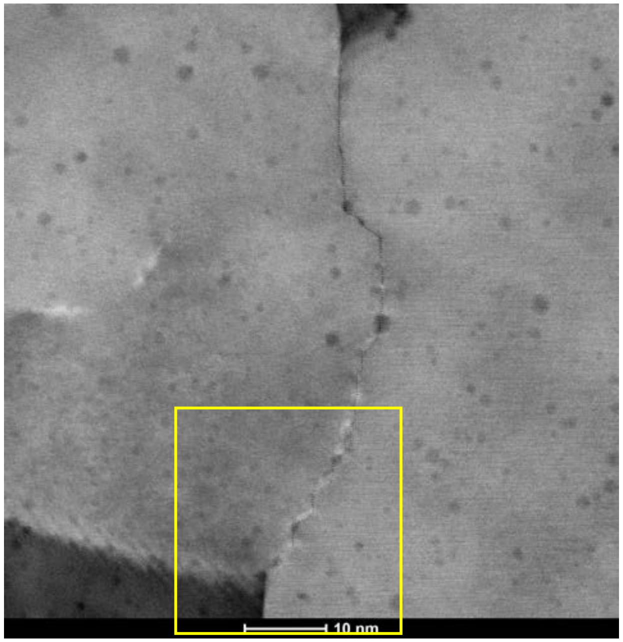

feature ID	x(nm)	y(nm)
fj19	41.03	49.20
fj20	40.54	49.50
fj21	40.56	52.12
fj22	38.53	53.31
fj23	38.48	56.05
fj24	36.19	57.21
fj25	36.10	59.84
fj26	33.94	61.15
fj27	33.89	63.90
fj28	31.66	65.21

Post-Irradiation

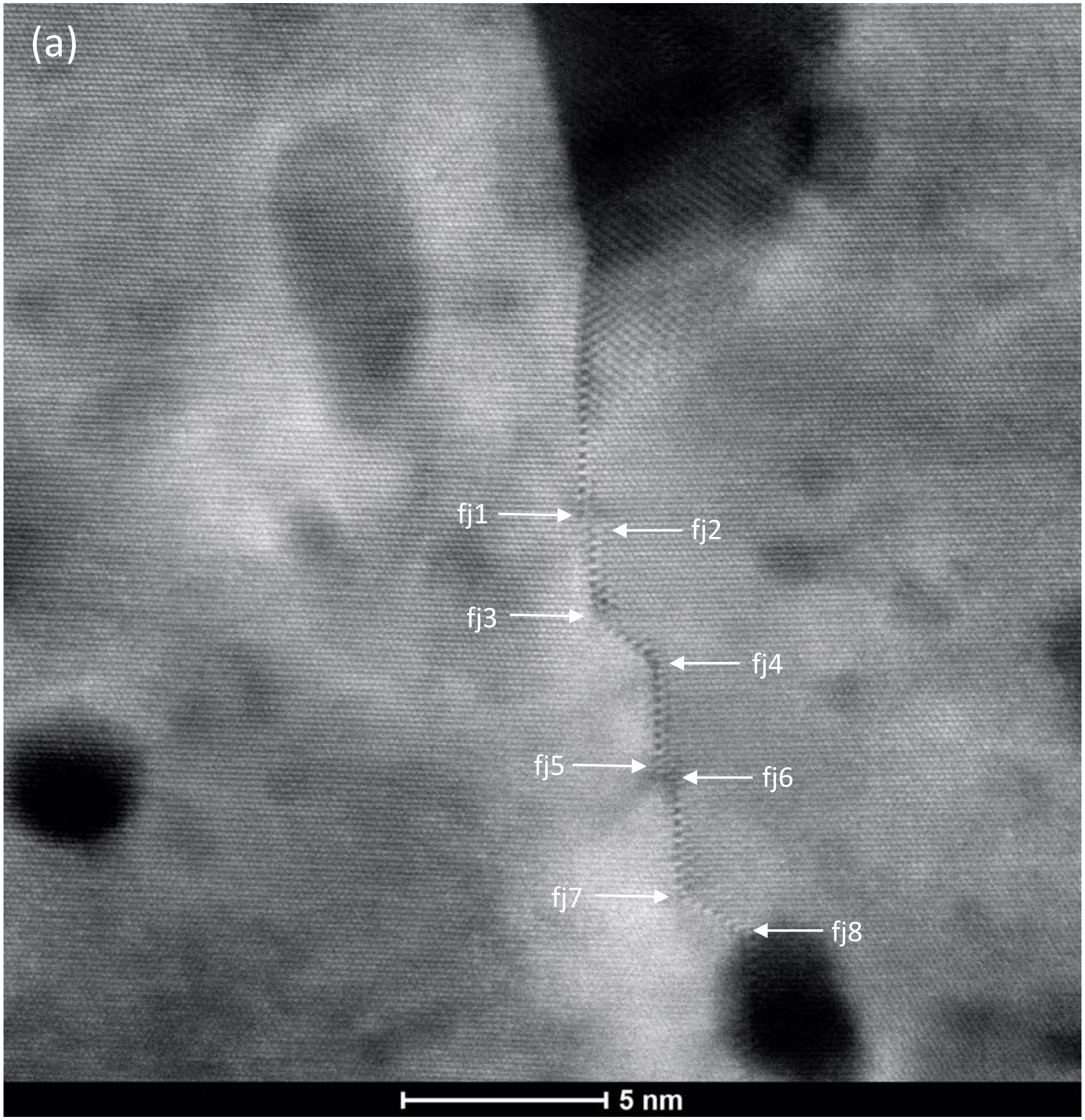

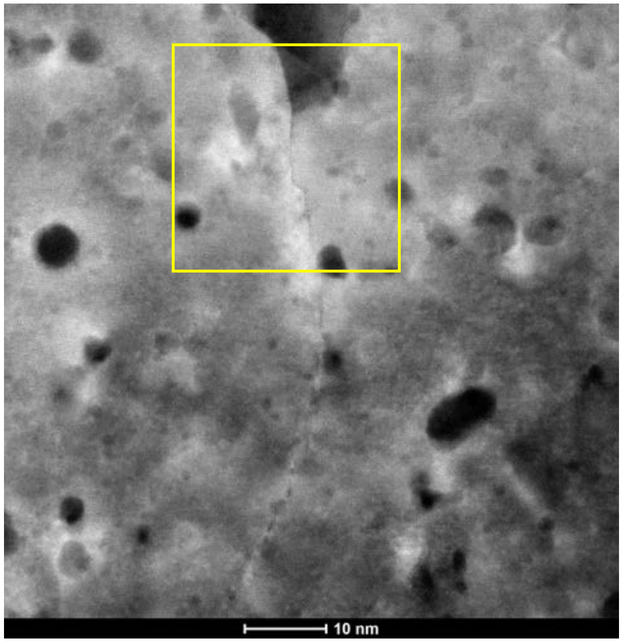

feature ID	x(nm)	y(nm)
fj1	33.39	18.60
fj2	33.73	19.06
fj3	33.77	20.91
fj4	35.21	21.98
fj5	35.33	24.78
fj6	35.68	24.98
fj7	35.73	27.55
fj8	37.46	28.63

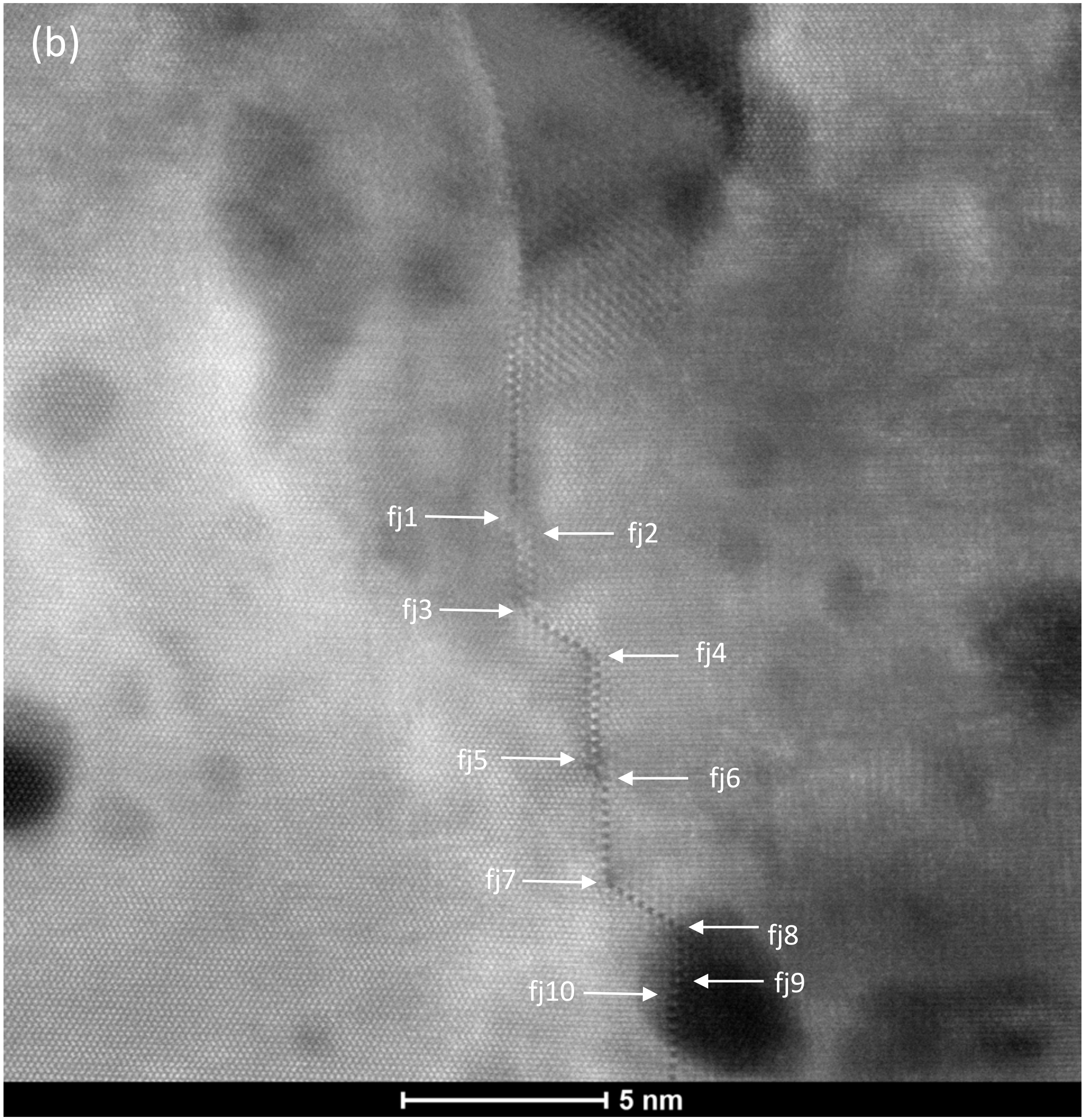

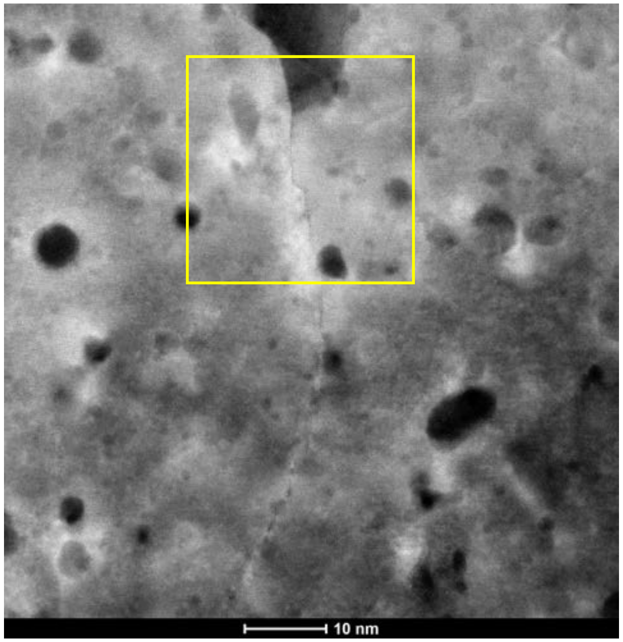

feature ID	x(nm)	y(nm)
fj1	33.41	18.79
fj2	33.77	19.15
fj3	33.72	20.99
fj4	35.36	22.14
fj5	35.32	24.53
fj6	35.66	24.88
fj7	35.62	27.49
fj8	37.46	28.52
fj9	37.43	29.65
fj10	37.21	29.98

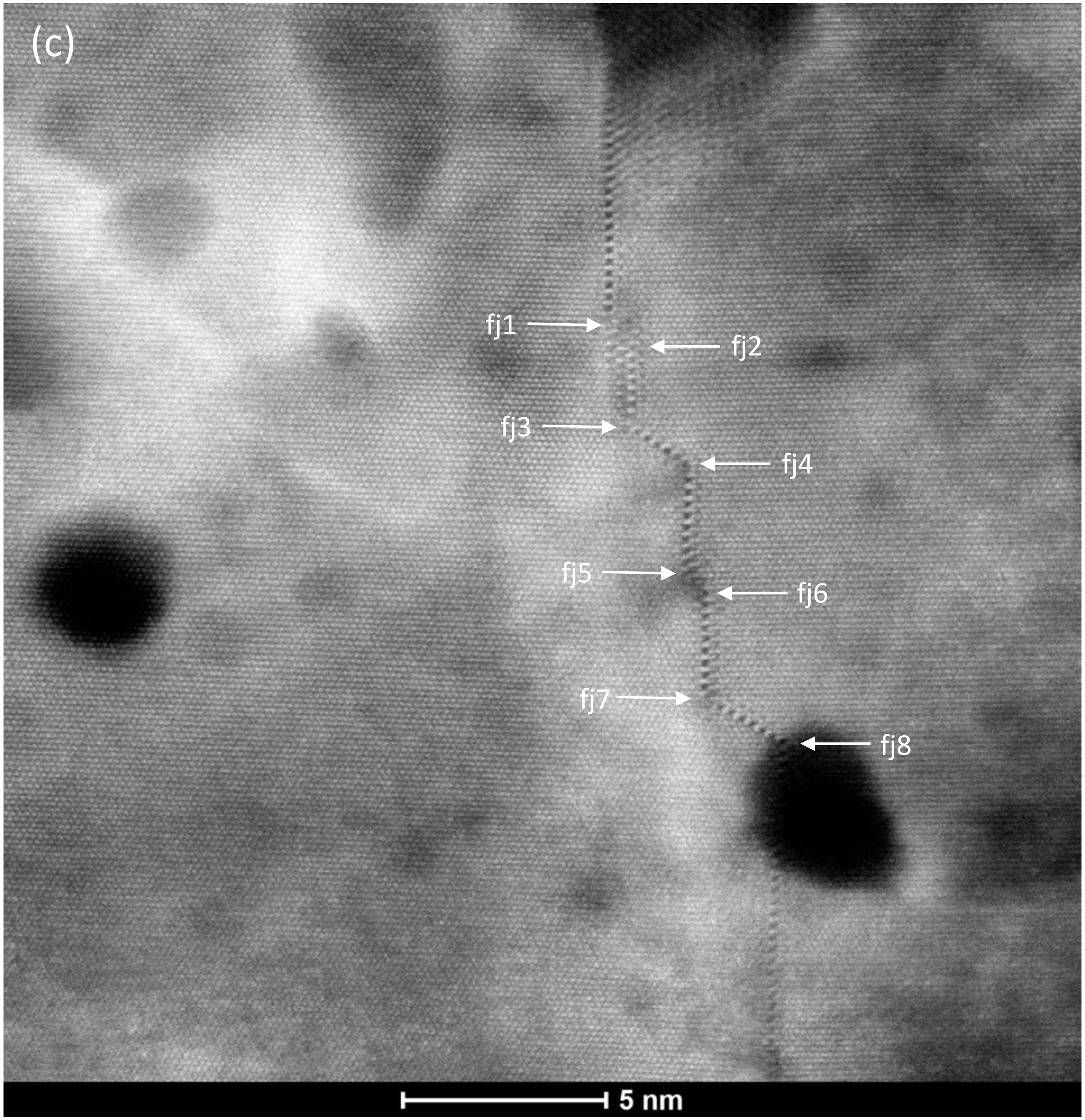

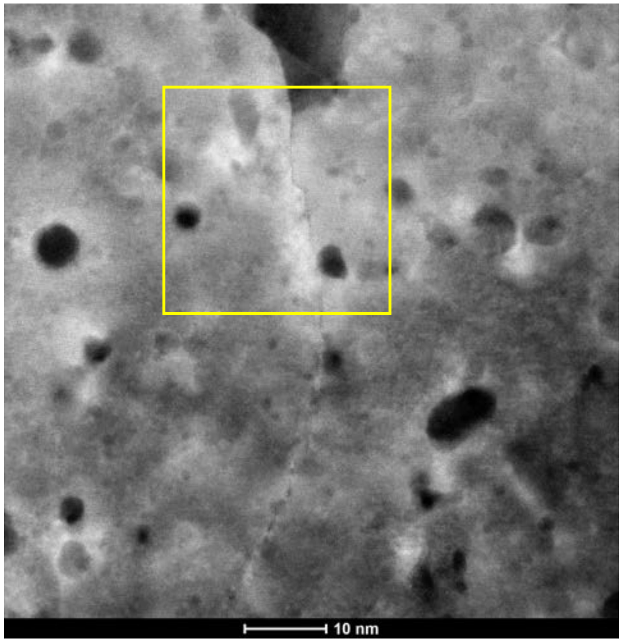

feature ID	x(nm)	y(nm)
fj1	33.34	18.60
fj2	33.89	19.02
fj3	33.72	21.07
fj4	35.28	22.00
fj5	35.23	24.60
fj6	35.70	25.13
fj7	35.64	27.45
fj8	37.52	28.62

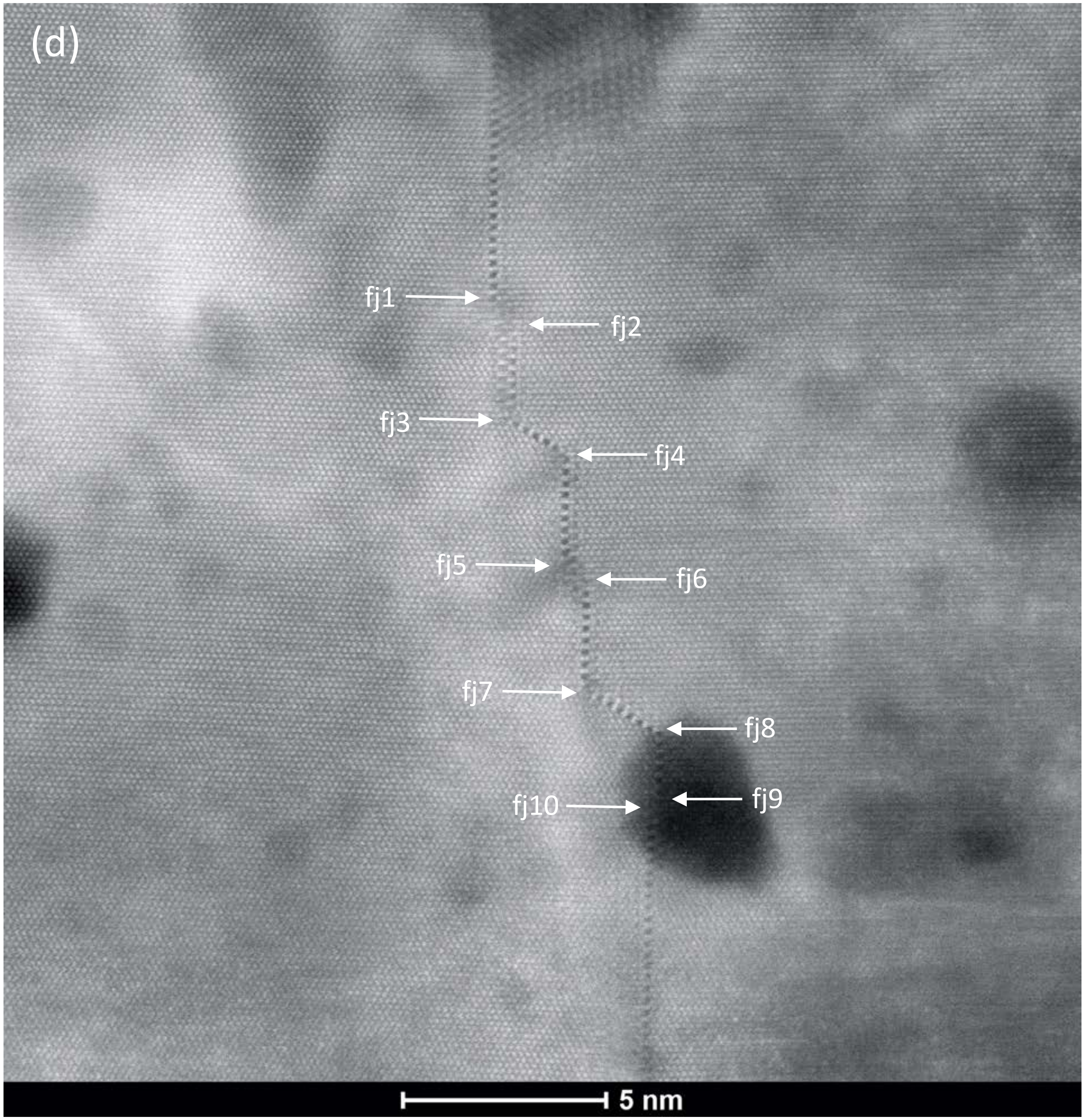

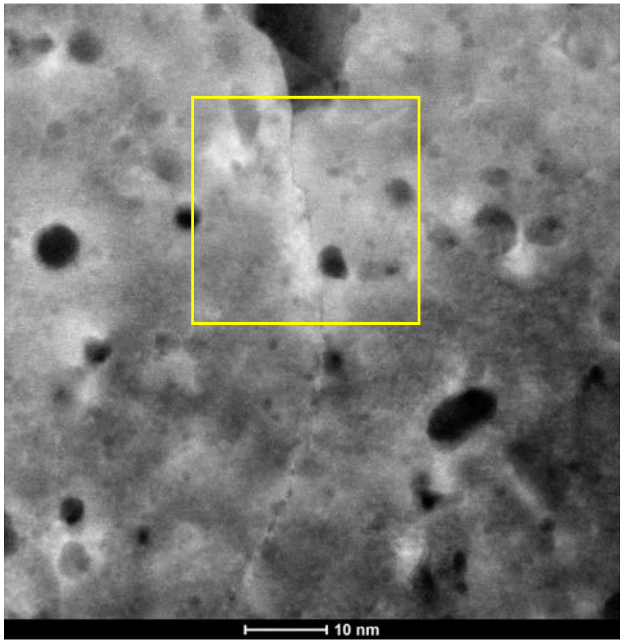

feature ID	x(nm)	y(nm)
fj1	33.48	18.39
fj2	33.91	18.95
fj3	33.76	21.14
fj4	35.22	22.05
fj5	35.15	24.48
fj6	35.70	25.20
fj7	35.66	27.54
fj8	37.43	28.72
fj9	37.41	30.34
fj10	37.25	30.45

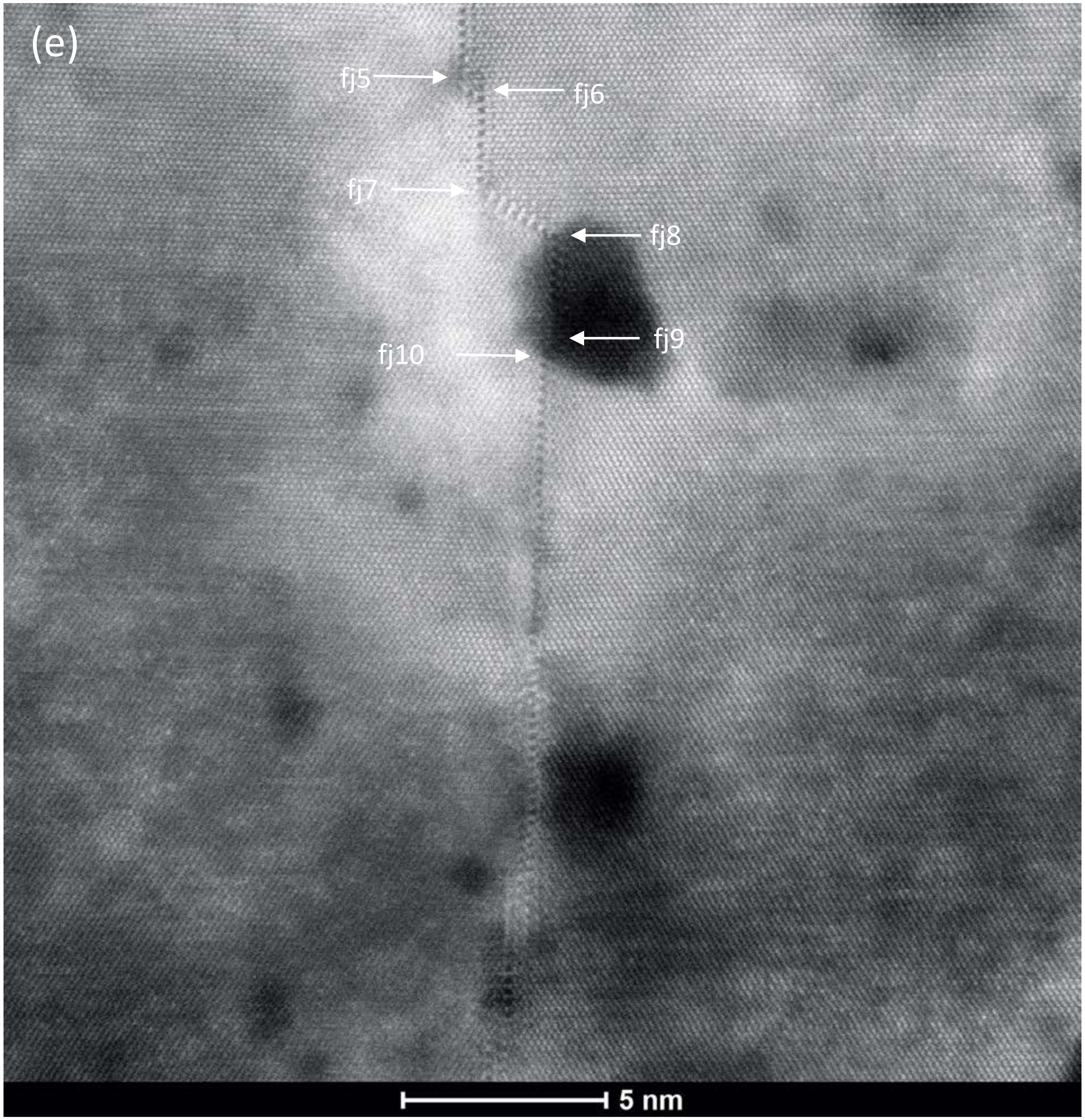

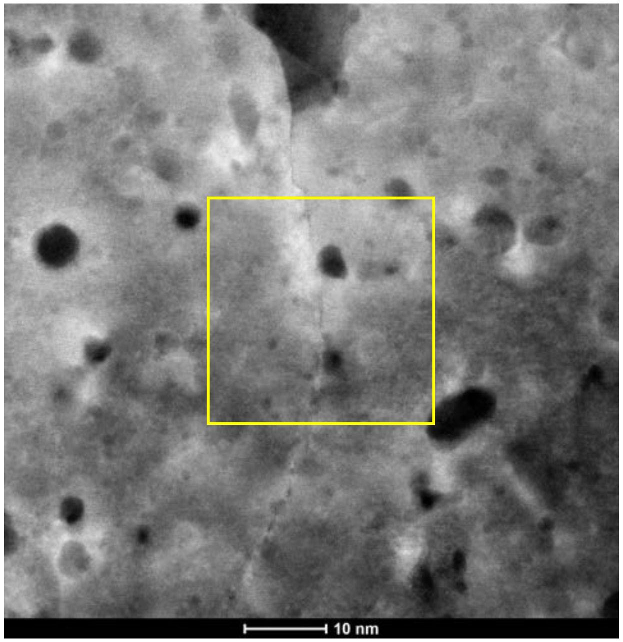

feature ID	x(nm)	y(nm)
fj5	35.27	24.73
fj6	35.75	25.03
fj7	35.69	27.53
fj8	37.48	28.61
fj9	37.43	30.03
fj10	37.29	30.41

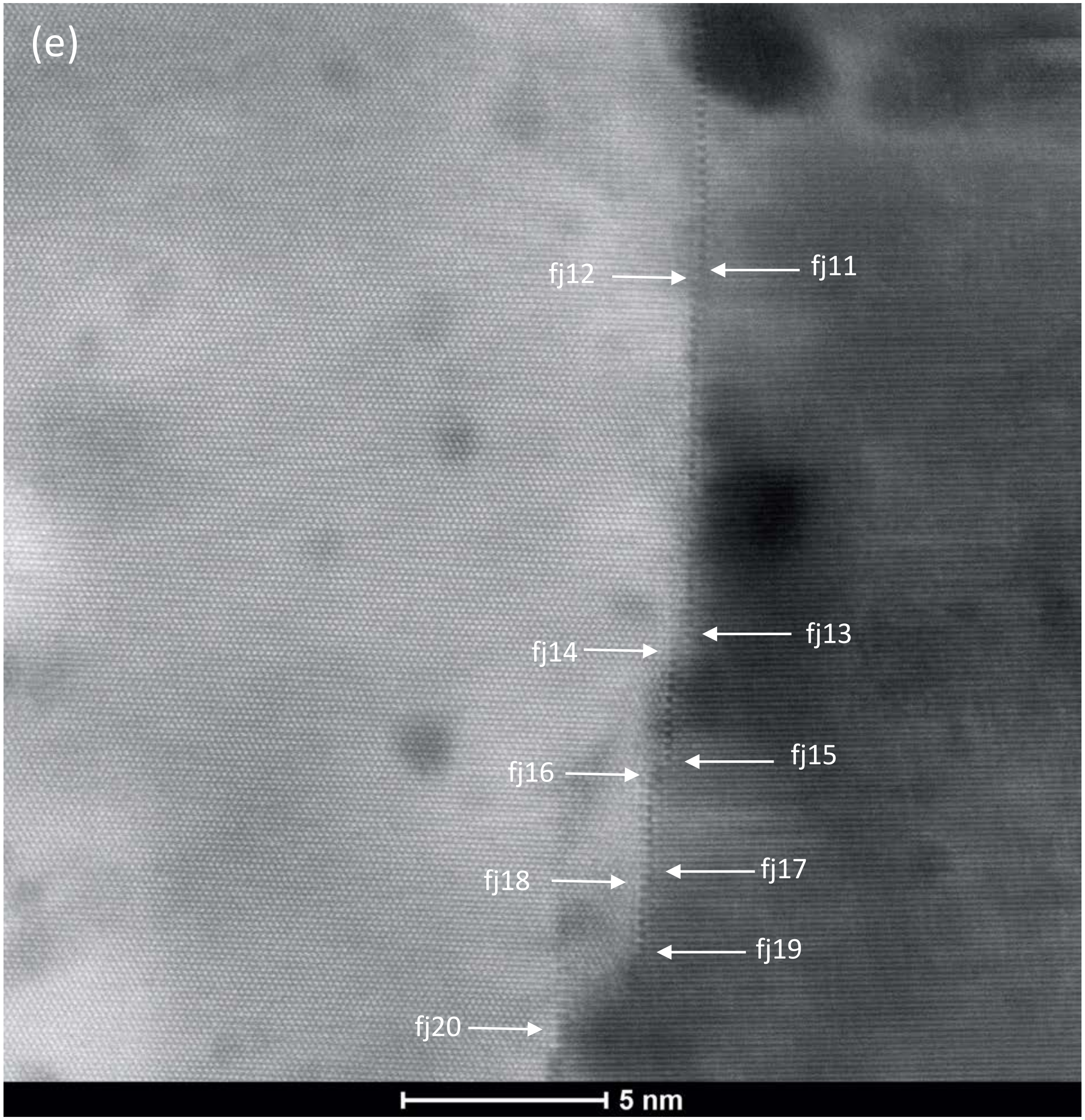

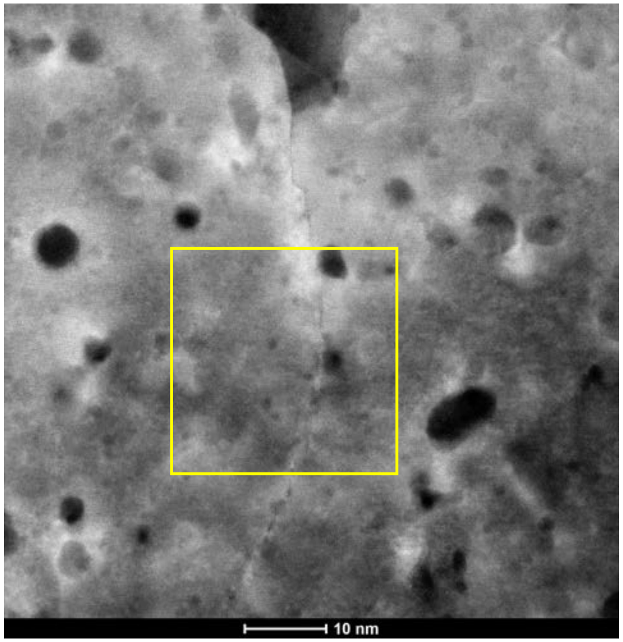

feature ID	x(nm)	y(nm)
fj11	37.07	36.07
fj12	36.87	36.42
fj13	36.71	44.96
fj14	36.40	45.10
fj15	36.26	47.67
fj16	35.87	48.05
fj17	35.87	50.42
fj18	35.71	50.53
fj19	35.62	52.27
fj20	33.71	53.95

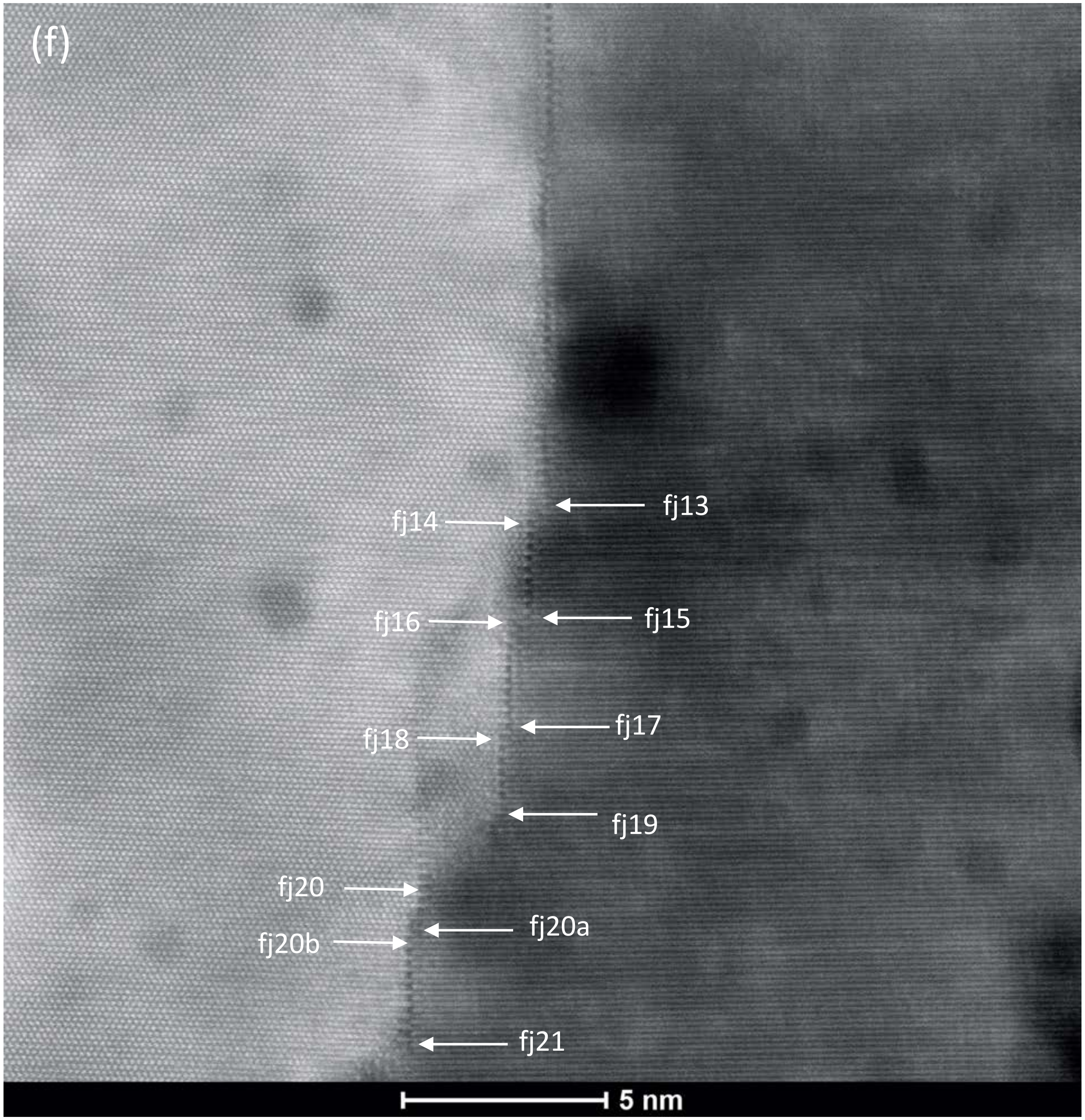

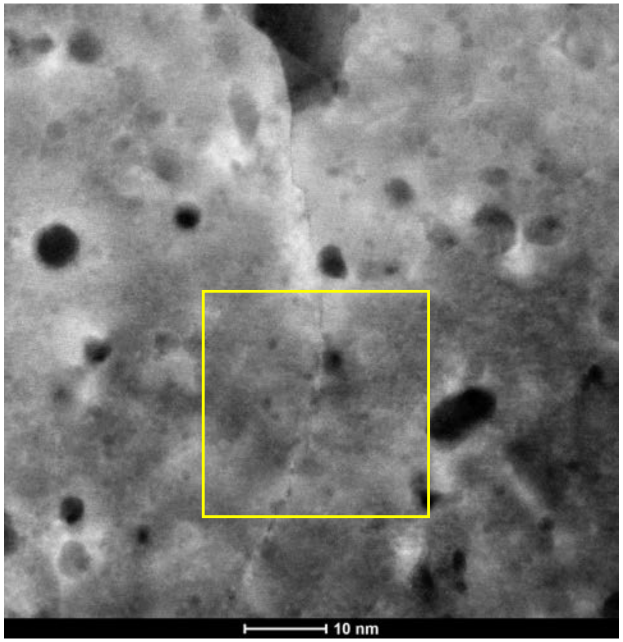

feature ID	x(nm)	y(nm)
fj13	36.67	44.85
fj14	36.31	45.01
fj15	36.25	47.73
fj16	35.86	47.91
fj17	35.81	50.34
fj18	35.63	50.56
fj19	35.66	52.50
fj20	33.77	53.95
fj20a	33.67	55.26
fj20b	33.43	55.38
fj21	33.42	57.61

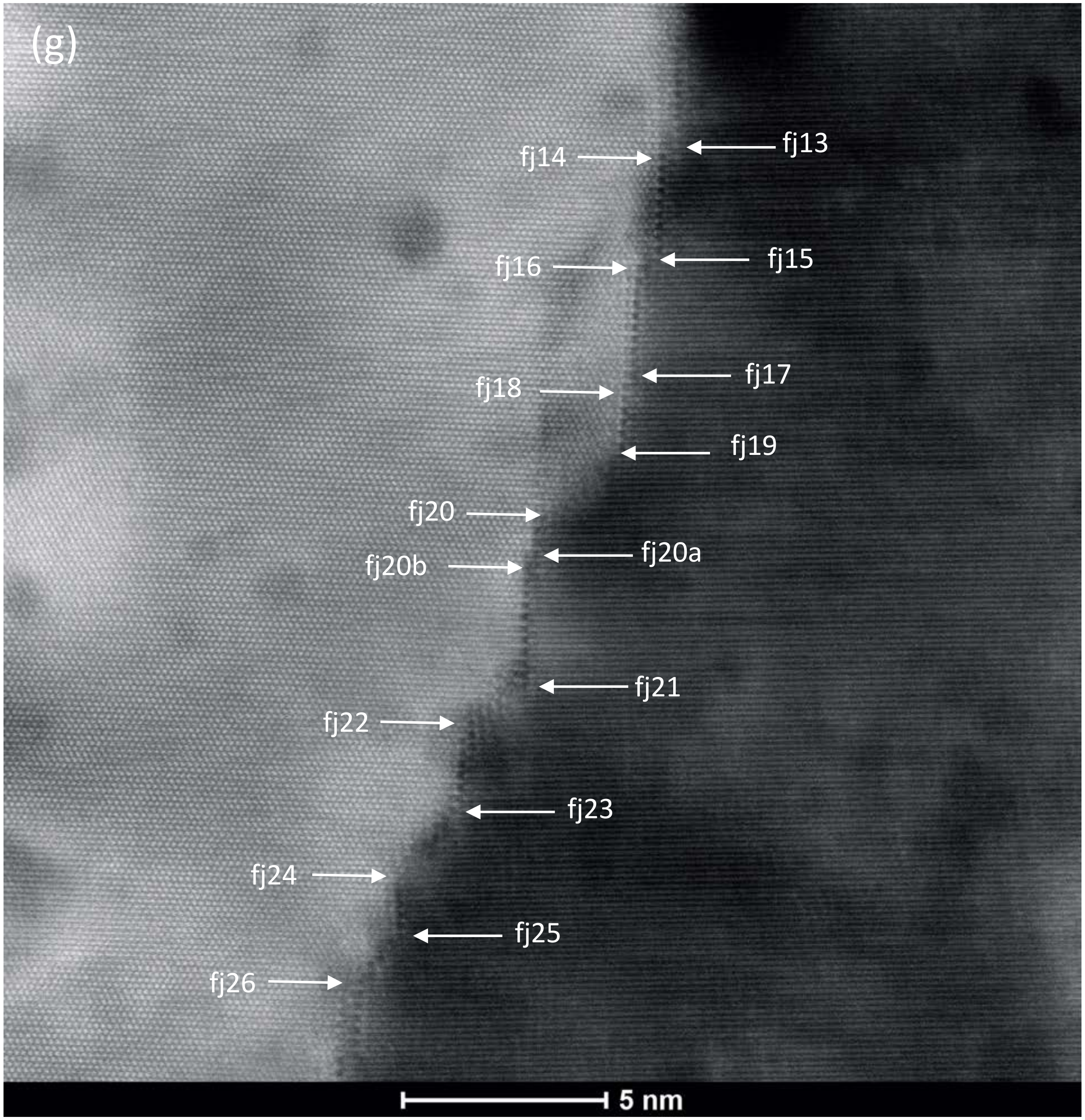

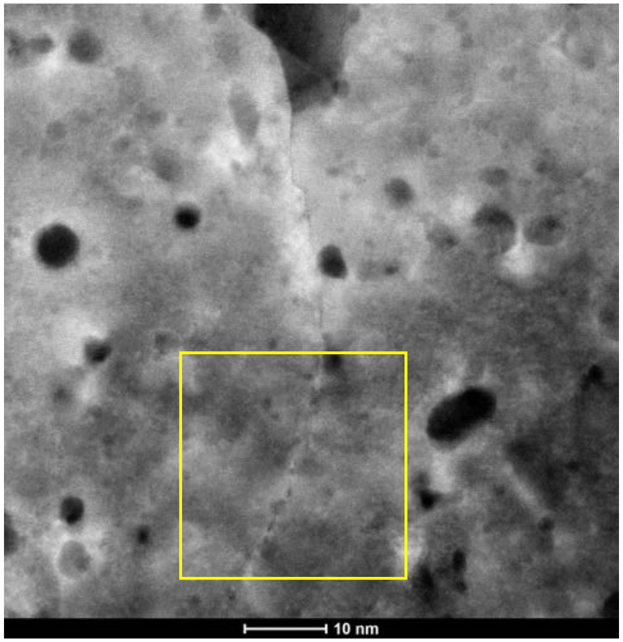

feature ID	x(nm)	y(nm)
fj13	36.92	44.52
fj14	36.61	44.89
fj15	36.49	47.37
fj16	36.01	47.78
fj17	35.90	50.34
fj18	35.71	50.63
fj19	35.61	52.10
fj20	33.60	53.85
fj20a	33.57	55.14
fj20b	33.36	55.28
fj21	33.36	57.67
fj22	31.90	58.79
fj23	31.79	60.42
fj24	30.34	62.15
fj25	30.40	63.64
fj26	29.39	64.78

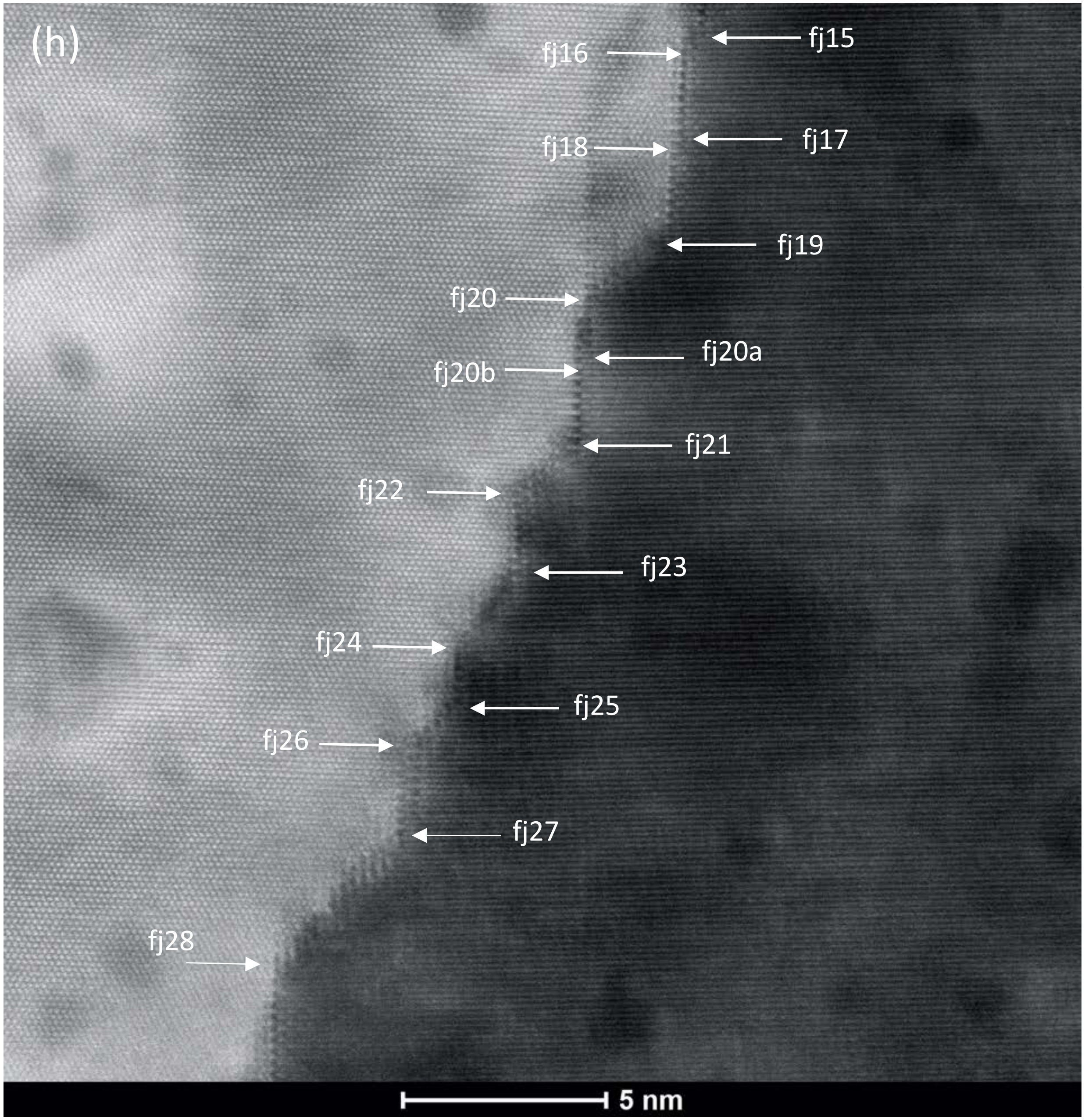

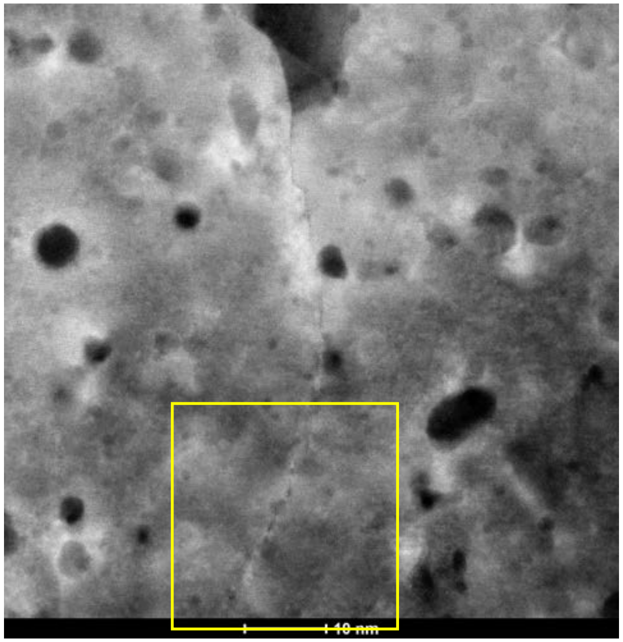

feature ID	x(nm)	y(nm)
fj15	36.27	47.68
fj16	35.83	48.07
fj17	35.74	50.33
fj18	35.56	50.42
fj19	35.51	52.35
fj20	33.56	54.07
fj20a	33.57	55.15
fj20b	33.33	55.25
fj21	33.24	57.70
fj22	31.82	58.67
fj23	31.92	60.37
fj24	30.44	62.20
fj25	30.46	63.57
fj26	29.32	64.70
fj27	29.28	66.46
fj28	26.16	69.90

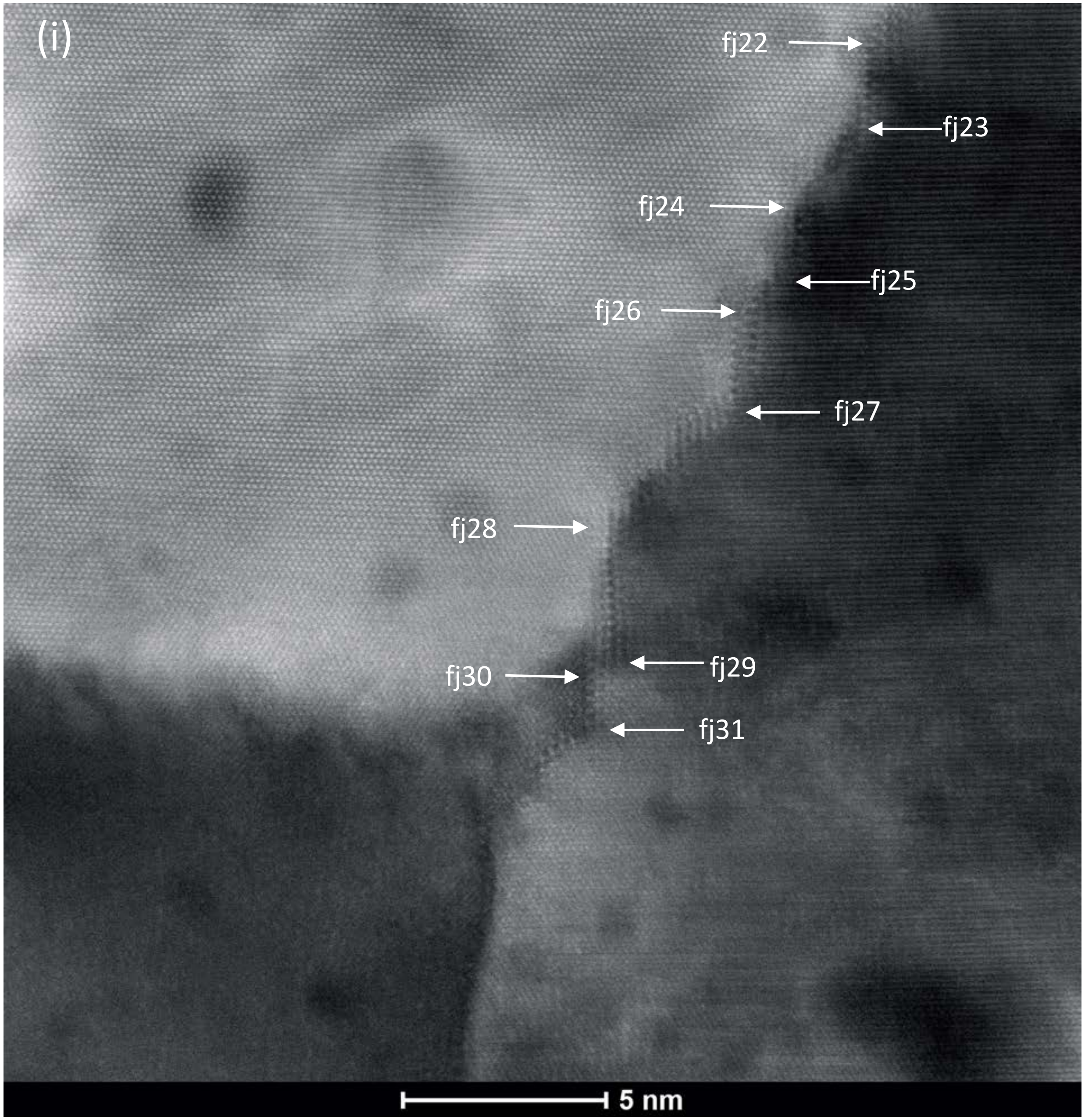

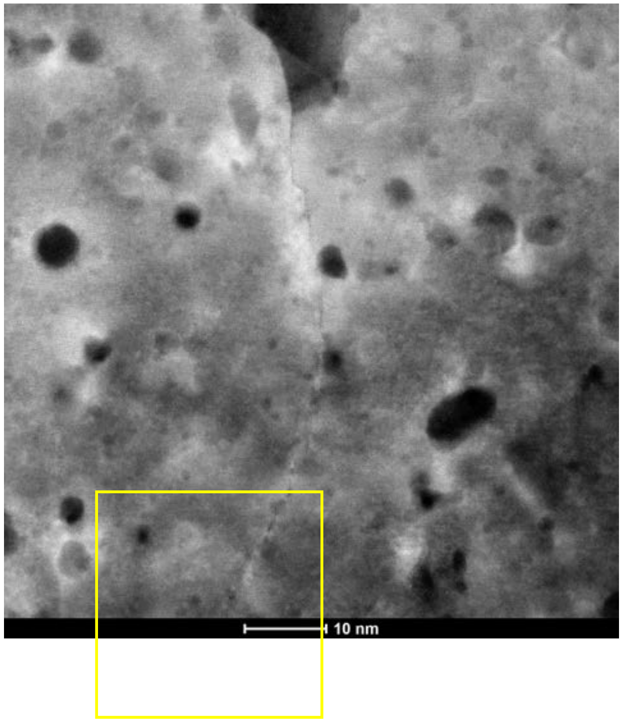

feature ID	x(nm)	y(nm)
fj22	32.05	58.39
fj23	31.97	59.94
fj24	30.35	62.32
fj25	30.25	63.62
fj26	29.22	64.87
fj27	29.10	66.74
fj28	25.85	69.84
fj29	25.80	73.17
fj30	25.32	73.54
fj31	25.37	75.02
